# Inflammatory biomarkers in cerebral venous thrombosis versus ischemic stroke: a network meta-analysis

**DOI:** 10.3389/fneur.2025.1634369

**Published:** 2025-11-26

**Authors:** Xinlong Shen, Ling Chen, Liling Shen, Shuling Chen, Shuwen Mu, Li Chen, Shousen Wang, Ziqi Li

**Affiliations:** 1 Department of Neurosurgery, Dongfang A liated Hospital of Xiamen University, School of Medicine, Xiamen University, Xiamen, Fujian, China; 2 Fujian Provincial Clinical Medical Research Center for Minimally Invasive Diagnosis and Treatment of Neurovascular Diseases, Fuzhou, China; 3 Department of Science and Education, 900th Hospital of PLA Joint Logistic Support Force, Fuzhou, Fujian, China; 4 Department of Epidemiology and Health Statistics, School of Public Health, Fujian Medical University, Fuzhou, Fujian, China; 5 Aberdeen Medical School, College of Life Sciences and Medicine, University of Aberdeen, Aberdeen, United Kingdom; 6 Department of Neurosurgery, Fuzhou University A liated Provincial Hospital, Fujian, China; 7 Department of Neurosurgery, Fuzong Clinical Medical College of Fujian Medical University, Fuzhou, Fujian, China

**Keywords:** cerebrovascular disorders, ischemic stroke, cerebral venous thrombosis, inflammation, inflammatory marker

## Abstract

**Background:**

In cerebrovascular diseases (CVD), the management strategies for ischemic stroke (IS) and cerebral venous thrombosis (CVT) have significant differences, but the underlying inflammation-driven mechanisms in these two conditions have not been fully translated into individualized intervention criteria.

**Methods:**

We searched PubMed, Embase, Web of Science and Cochrane Library through February 1, 2025, and included 18 eligible studies in a Bayesian network meta-analysis following PRISMA-NMA. The data were processed using Revman (version 5.4.1) and R (version 4.3.3). The Grading of Recommendations, Assessment, Development and Evaluation (GRADE) method was used to assess the quality of evidence. This study was registered in PROSPERO (CRD42024539498).

**Results:**

In total, 18 studies were included in the review. The results showed that acute-phase inflammatory markers were significantly elevated in both CVT and IS. CVT was associated with a relatively stronger systemic inflammatory response, while lymphocyte counts were reduced in both, suggesting a immunosuppressive phenomenon in cerebral thrombotic disease. This network Meta-Analysis showed that CRP (MD = 7.58, 95% CI: 2.48–14.09) and IL-6 (MD = 6.98, 95% CI: 2.75–11.44) were more significantly elevated in the acute phase in CVT patients than in IS patients, suggesting they could serve as key inflammatory markers for differentiating the two conditions.

**Conclusion:**

Inflammatory markers exhibit both specific differences and shared characteristics in CVT and IS. CRP and IL-6 were higher in CVT than in IS in Bayesian NMA, suggesting potential adjunctive markers for differential diagnosis; however, these findings are hypothesis-generating and require prospective validation, and neuroimaging remains the diagnostic gold standard.

**Systematic review registration:**

https://www.crd.york.ac.uk/PROSPERO/view/CRD42024539498, CRD42024539498.

## Introduction

Cerebrovascular diseases (CVD) are major causes of disability and mortality worldwide; two major types are ischemic stroke (IS) and cerebral venous thrombosis (CVT). Although both are characterized by vascular occlusion (blood vessel blockage), their pathological mechanisms, risk factors, and clinical management have significant difference.

IS is caused mainly by the rupture of atherosclerotic plaques or cardiac embolism leading to cerebral blood flow interruption. It is the second leading cause of death and disability worldwide, with an annual incidence of 200 per 100,000, of which more than 75 per cent occurs in people over 60 years of age (1). Recent studies have revealed that ischemia–reperfusion injury activates microglia through the TLR4/NF-κB pathway, releasing proinflammatory factors such as IL-1β and TNF-α, which drive microglia toward proinflammatory (M1-type) polarization, resulting in an “inflammation-thrombosis vicious cycle” (2, 3). Although antithrombotic therapy reduces the acute-phase mortality rate, survivors still have a risk of recurrence, suggesting that the chronic inflammation-driven pathological process requires clinical intervention.

Unlike IS, CVT is a rare cerebrovascular disease of the intracranial venous system (annual incidence of about 1.3/100,000 people), which affects mainly young women (the male-to-female ratio is 1:3) and is characterized by endothelial damage to the venous sinuses, hypercoagulability, and stagnant blood flow (4, 5). The thrombi in CVT are fibrin-rich and their formation is closely related to the activation of the coagulation cascade driven by the monocyte-tissue factor (TF) axis and the inflammatory response mediated by the interleukin (IL)-6/STAT3 pathway (6). Although anticoagulation significantly affects prognosis, many patients may experience chronic symptoms such as cognitive impairment or headache after treatment, possibly related to a persistent low inflammatory response (e.g., mildly elevated IL-6 and CRP) (5).

Although both IS and CVT involve “inflammation-thrombosis interaction” pathways, their specific mechanisms are significantly different (Table 1). Commonalities: Systemic inflammatory responses drive endothelial injury and thrombus expansion, forming a vicious cycle. Differences: In IS, arterial thrombosis with inflammation concentrated in the ischaemic region. In CVT, venous thrombosis is associated with more a more extensive inflammatory response and a high risk of chronicity.

**Table 1:** Comparing the characteristics of CVT and IS.

Characteristics	CVT	IS
Incidence rate	1.3/100,000 people (mainly in young women)	200/100,000 people (mainly in middle-aged and elderly people)
Thrombus type	Red thrombus (fibrin-rich)	White thrombus (mainly platelet aggregation)
Main inflammatory response	Neutrophil-NETs axis Monocyte-TF axis IL-6/STAT3 pathway	Microglia-TLR4/NF-κB
Treatment focus	Anticoagulation and inhibition of acute thrombotic inflammation.	Antithrombotic therapy and regulation of chronic arterial inflammation.

CVT, Cerebral Venous Thrombosis; IL-6, Interleukin-6; IS, Ischemic Stroke; NETs, Neutrophil Extracellular Traps; NF-κB, Nuclear Factor kappa-light-chain-enhancer of activated B cells; STAT3, Signal Transducer and Activator of Transcription 3; TF, Tissue Factor; TLR4, Toll-like Receptor 4.

Currently, in clinical practice, the diagnosis of these two diseases relies mainly on imaging evidence. However, it often faces issues such as insufficient sensitivity or delayed identification. Therefore, clarifying the differences in specific inflammatory markers between the two types of diseases is valuable for early differential diagnosis, targeted anti-inflammatory therapy and prognostic stratification. By systematically comparing their inflammatory marker profiles, this study aims to: reveal the differences in inflammatory marker profiles between IS and CVT, screen for disease-specific markers, and provide a low-cost diagnostic tool for resource-limited areas.

## Methods

### Reporting guidelines

This study followed the structure of the Preferred Reporting Items for Systematic Review incorporating Network Meta-analysis (PRISMA-NMA) (7). The study protocol was registered in advance with PROSPERO (CRD42024539498).

### Search strategies

Several literature databases were searched in this study, namely PubMed, Embase, Web of Science and the Cochrane Library for publications published before February 2025. The keywords for the database searches included “cerebral venous thrombosis,” “cerebral venous sinus thrombosis,” “ischemic stroke,” “stroke,” “interleukin-6,” “C-reactive protein,” “high-sensitivity C-reactive protein,” “systemic immune-inflammation index,” “neutrophil to lymphocyte ratio,” “platelet to lymphocyte ratio,” “monocyte to high-density lipoprotein ratio” (the full search strategy can be found in Supplementary Table S1). The references of the retrieved articles were then thoroughly reviewed for additional reports that we may have missed in the search. The grey literature (e.g., conference abstracts, clinical trial registries) retrieved from the databases was not included, as it did not meet the predefined inclusion criteria for this network meta-analysis.

### Study selection criteria

Two-stage screening by two reviewers; disagreements resolved by consensus/third reviewer；data-extraction double-entry. The enrolled studies included met the following criteria: (1) patients with a definitive diagnosis of IS or CVT; (2) inflammatory markers expressed as the means ± standard deviate (SD) or as median with interquartile range (IQR); (3) comparisons among IS patients, CVT patients and healthy controls. The exclusion criteria included: (1) patients with complications due to venous thrombosis or stroke, such as renal veins, mesenteric veins, stroke-associated pneumonia and poststroke depression; (2) inflammatory markers reported as median with range, median with 95% confidence interval (95%CI), mean with range or 95%CI, or as effect estimates (odds ratio and relative risk); (3) CVT or IS patients with transient risk factors (such as recent surgery, trauma, fracture, estrogen therapy, or newly diagnosed atrial fibrillation); (4) studies presenting data in non-extractable formats (e.g., only qualitative descriptions, non-numeric figures without raw values) or with incomplete outcome data that preclude valid statistical analysis.

### Data extraction

The relevant data were extracted from each article via structured forms. The following information was retrieved for each article: study design, demographics, inflammatory markers, blood sample collection time and diagnosis time. The available markers included white blood cells (WBC), neutrophils, lymphocytes, monocytes, platelet-lymphocyte ratio (PLR), neutrophil-lymphocyte ratio (NLR), high-sensitivity C-reactive protein (hs-CRP), C-reactive protein (CRP), IL-6, systemic immune inflammation index (SII), neutrophil-lymphocyte ratio (NLR), platelet-lymphocyte ratio (PLR), monocytes-High-density lipoprotein ratio (MHR). Data were collected by four reviewers (X. Shen, L. Chen, L. Shen, and S. Chen), and if inconsistency existed between the two reviewers, the two other reviewers would re-examined the data and made a final decision based upon the majority.

### Data pre-processing

Before conducting in-depth statistical analyses, rigorous preprocessing was performed on all biomarker data. Data cleaning involved outlier detection and normality testing of the data. The unit conversion of biomarkers (e.g., conversion of white blood cell counts to *10^9/L) was also performed at this stage to facilitate subsequent statistical analyses.

### Descriptive statistics

Descriptive statistics were calculated for each inflammatory marker indicator in this study. The mean, standard deviation, median and interquartile range were calculated for each indicator. This provided the necessary overview and distribution of data for meta-analysis.

### Data conversion and consolidation analysis

The mean (SD) was used for the pooled analysis, and the median (IQR) was converted to the mean (SD) for the meta-analysis in this study. Inflammatory marker data often exhibit non-normal distributions. To estimate the sample mean and standard deviation (SD) from medians and interquartile ranges (IQRs), we applied the Box-Cox (BC) transformation method proposed by McGrath et al. (8). The core assumption of this method can be summarized as follows: By optimizing the parameter *λ* to transform the data into a normal distribution, where λ is selected based on the quantile symmetry criterion (e.g., *fλ*(*Q*3) − *fλ*(*Q*2) = *fλ*(*Q*2) − *fλ*(*Q*1)), and inverting the original scale parameters are inverted via Monte Carlo simulation (8). This method demonstrates strong robustness for moderately skewed data (e.g., non-normally distributed data such as inflammatory marker date). Only medians with IQRs were converted to means and SDs using the McGrath Box-Cox approach; studies reporting medians with ranges or 95% CIs were not converted and were excluded from quantitative synthesis.

### Data synthesis and analysis

All the statistical analyses were conducted via Review Manager (version 5.4.1) and R (version 4.3.3) in this study. The remaining variables were expressed as mean difference (MD), 95% confidence interval (CI), and dichotomous variables were expressed as OR, 95% CI to derive outcome statistics. A Bayesian network meta-analysis was performed to synthesize evidence across comparisons, with effect sizes reported as mean differences (MD) and 95% credible intervals (CrI). Retrospective case–control studies and prospective/retrospective cohort studies were be included in this study. To address potential variation among different types of studies, we used the Q - test (chi - square test) to assess data heterogeneity. If the *I^2^* statistic was less than 50% of the value then there was no significant heterogeneity, justifying the use of a fixed-effects model for the calculation of the combined effect sizes; otherwise, a random-effects model was applied (9). To evaluate the stability of the statistical results, sensitivity analyses were conducted by excluding individual studies one by one to assess changes in the pooled effect size. The absence of significant alterations in the pooled effect size following the exclusion of any single study indicated good stability of the analysis results. For traditional meta-analysis outcomes, publication bias was visually inspected via funnel plots; for network meta-analysis outcomes, publication bias was evaluated via adjusted contrast funnel plots and Egger’s regression test (10). We conducted a network meta-analysis via Bayesian hierarchical modelling (“gemtc” package of R) to make pairwise comparisons between CVT, IS and controls.

### Risk of bias assessment and quality of outcomes assessment

The Newcastle-Ottawa Scale (NOS) and was employed in this meta-analysis to assess the quality of non-randomized trials (11). Scores of 7–9, 4–6, and 4 were classified as having a low, moderate, or high risk of bias, respectively. In addition, the Risk of Bias in Individual Studies—Extended (RoB-I/E) tool was used to evaluate the risk of bias across seven domains (D1–D7) for all included studies, with each domain rated as low, some concerns, or high risk, and an overall risk of bias determined accordingly (12). The Grading of Recommendations, Assessment, Development and Evaluation (GRADE) guidelines for systematic reviews and network meta-analyses were followed to assess the quality of outcomes (13).

### Certainty assessment

For assessing imprecision, a mean difference threshold of ± 0.20 was considered clinically significant (14), which was also applied to heterogeneity, and the consistency of direct and indirect effects was evaluated against this threshold to assess incoherence in order to validate the consistency of the results of the different comparison pathways and to ensure the reliability of the network evidence.

## Results

### Literature characteristics

**Figure 1:**
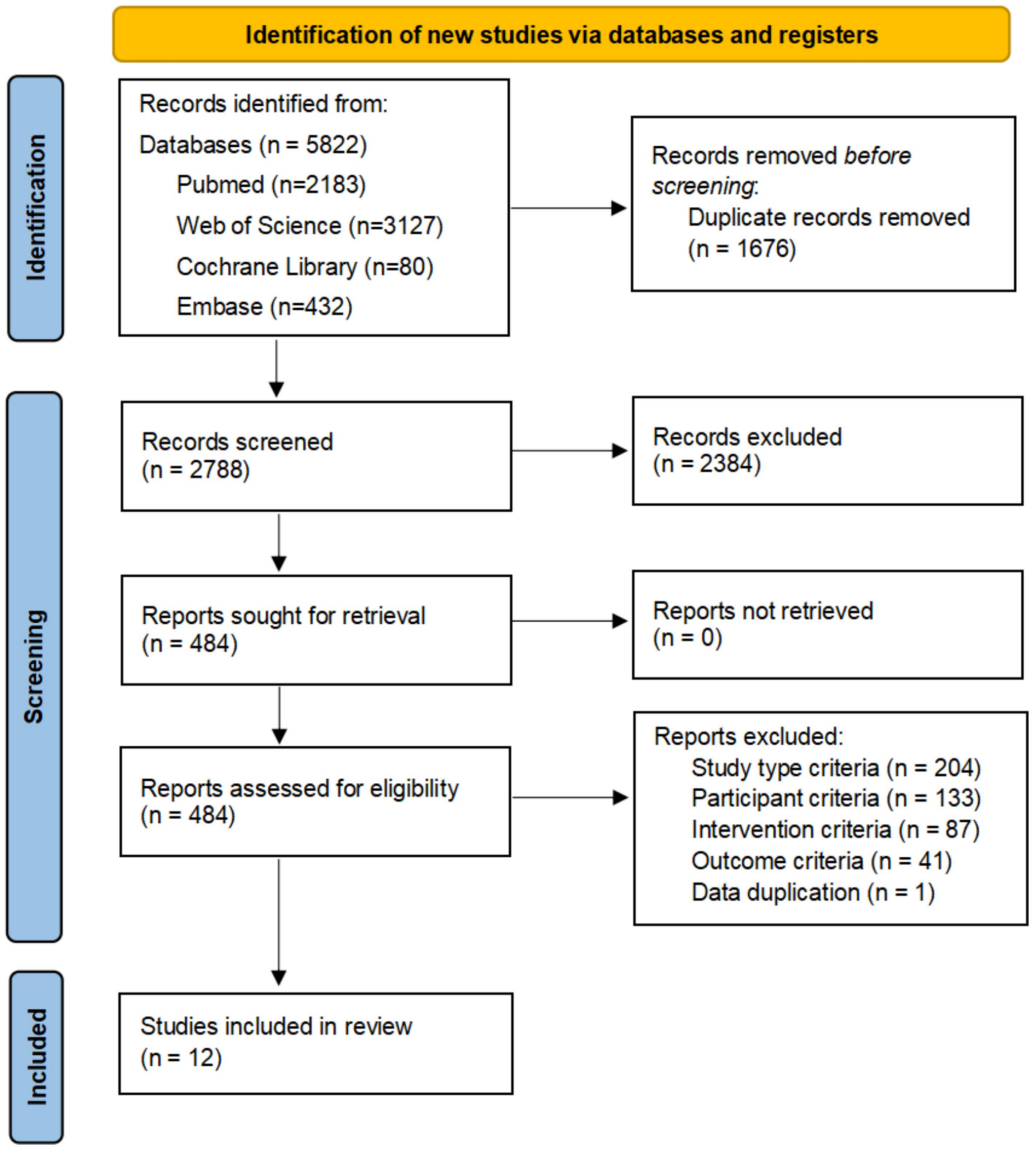
Flowchart of all studies identified, included and excluded in this systematic review and meta-analysis. Initially, 5,822 records were identified, with 1,676 duplicates removed, leaving 2,788 records screened. Of these, 2,384 were excluded, 484 reports sought and assessed for retrieval. No reports were lost. Exclusions included criteria like study type (204), participant (133), intervention (87), outcome (41), and data duplication (1). Twelve studies were included in the review.

From the initial 5,822 records identified, 1,676 duplicates were removed. The remaining 4,146 records were screened for title and abstract, and then 484 selected articles were reviewed in full text. Ultimately, 18 articles were eligible for inclusion in this study (15–32). Study selection is summarized in the PRISMA 2020 flow diagram (Figure 1). The characteristics of the studies included are summarised in Table 2.

**Table 2:** The characteristics of the involved studies.

Author, year	Design (database)	CVT/IS cases (n)	Controls (n)	Blood indexes measured time and diagnosis time
Akboga et al. 2017 (15)	Retrospective case–control	Patients with CVT (80)	Individuals without CVT (197)	Blood samples were collected at admission and the time of CVT diagnosis was unknown.
Tekesin and Tunç 2019 (23)	Prospective case–control	Inpatients with CVT (36)	Healthy individuals without CVT (40)	Blood sample collection and CVT diagnosis took place at admission
Zhang et al. 2021 (26)	Retrospective case–control	Patients were newly diagnosed CVT (90)	Primary headaches as controls (60)	Blood tests were performed within 24 h of admission, CVT diagnosis took place at admission
Kamisli et al. 2012 (30)	Retrospective case–control	Patients with CVT (35)	Healthy individuals (27)	Blood samples were obtained before CVT diagnosis
Wang et al. 2018 (25)	Retrospective case–control	Inpatients with CVT (95)	Inpatients without CVT (41)	CVT was newly diagnosed and blood samples were obtained at admission.
Kula et al. 2024 (27)	Retrospective case–control	Patients diagnosed with CVST (40)	Healthy individuals without CVST (40)	Blood sample collection and CVT diagnosis took place at admission
Ding et al. 2023 (18)	Retrospective case–control	Patients diagnosed with CVST (146)	Inpatients without CVST (93)	CVT was newly diagnosed and blood samples were obtained after admission 1 to 2 days.
Kucukceran et al. 2022 (22)	Retrospective Cohort	patients diagnosed with CVST (57)	Inpatients without CVST (251)	Blood sample collection and CVT diagnosis took place at admission
Weng et al. 2021 (28)	Retrospective case–control	Patients diagnosed with AIS (216)	Healthy individuals without AIS (875)	Blood samples were collected within 24 h after admission and IS diagnosis took place at admission
Jenny et al. 2019 (20)	Case-cohort study design	Participants with incident IS (557)	Healthy individuals without IS (951)	Pre-stroke diagnosis,
Chang et al. 2005 (17)	Retrospective case–control	Patients diagnosed with IS (68)	Inpatients without IS (41)	Blood samples were collected within 48 h after admission and IS diagnosis Within 48 h after stroke onset
Miwa et al. 2013 (29)	Prospective Cohort Study	Patients were newly diagnosed IS (25)	Inpatients without IS (439)	Blood sample collection when IS diagnosis
Cai et al. 2021 (16)	Retrospective cohort study	Patients diagnosed with AIS (266)	Healthy individuals without AIS (2196)	Blood sample collection and AIS diagnosis took place at admission
Gao et al. 2021 (19)	Retrospective case–control	Patients diagnosed with AIS (283)	Healthy individuals without AIS (872)	Blood sample collection and AIS diagnosis took place at admission
Korkut et al. 2022 (21)	Prospective case–control study	Patients diagnosed with AIS (53)	Healthy individuals without AIS (41)	Blood sample collection and AIS diagnosis took place at admission
Tekesin et al. 2023 (24)	Retrospective case–control	Patients were newly diagnosed IS (70)	Healthy individuals without IS (70)	Blood sample collection and AIS diagnosis took place at admission
Gencdal et al. 2024 (31)	Retrospective case–control	Patients with AIS (124)	Healthy controls (126)	Blood sample collection and AIS diagnosis took place at admission
Liu et al. 2020 (32)	Retrospective case–control	Patients with IS (253)	Healthy controls (211)	Blood sample collection and IS diagnosis took place at admission

AIS, acute ischemic stroke; IS, ischemic stroke; CVST, cerebral venous sinus thrombosis; CVT, cerebral venous thrombosis.

### Quality assessment

**Figure 2:**
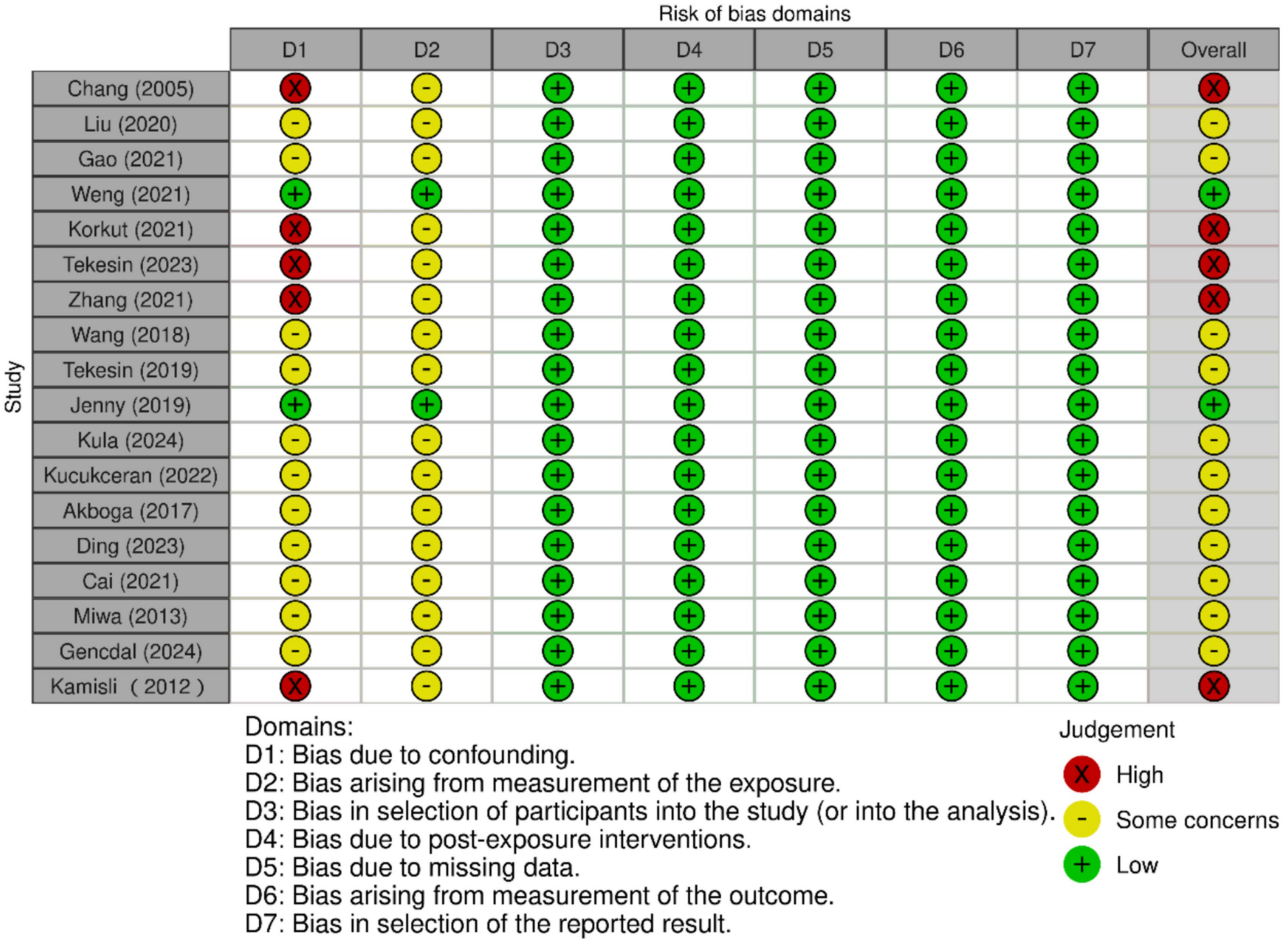
The forest plot of the risk of bias assessment for included studies. Columns labeled D1 to D7 represent different bias domains. Each cell uses traffic light symbols: red (X) for “High” risk, yellow (circle) for “Some concerns,” and green (plus) for “Low” risk. Studies are listed by author and year. Overall ratings are on the right, with a mix of judgments. A key below explains the symbols and domains.

The NOS was used to assess the risk of bias for the 18 included studies. Two reviewers independently scored each study, with discrepancies resolved through discussion. Fourteen studies (77.8%) were rated as low risk of bias, and four (22.2%) as moderate risk, with no studies classified as high risk. Detailed scores for the “selection,” “comparability,” and “outcome/exposure” domains, along with total scores, are presented in Table 3. In addition, the RoB-I/E assessment showed that the majority of studies had low or moderate risk of bias, with only five studies rated as high risk. Risk-of-bias domains (D1–D7) are defined in the Figure 2. Most studies performed well across randomization and other bias domains, although some studies had limitations in randomization or allocation concealment (17, 21, 24, 26, 30). Overall, the included studies presented an acceptable risk of bias, which should be considered when interpreting the results.

**Table 3:** Newcastle-Ottawa Scale for risk of bias assessment of the included studies (scores ≥ 7–9, 4–6, <4 are considered low, intermediate, and high risk, respectively).

No.	First author	Year	Selection	Comparability	Outcome/Exposure	Overall
1	Weng	2021	***	**	**	7*
2	Jenny	2019	****	**	**	8*
3	Chang	2005	***	*	**	6*
4	Miwa	2013	****	**	***	9*
5	Liu	2020	****	**	**	8*
6	Cai	2021	****	**	**	8*
7	Gao	2021	****	**	**	8*
8	Korkut	2021	**	*	***	6*
9	Tekesin	2023	**	*	***	6*
10	Zhang	2021	***	*	**	6*
11	Wang	2018	****	**	**	8*
12	Tekesin	2019	****	*	**	7*
13	Kamisli	2012	***	**	***	8*
14	Kula	2024	****	**	***	8*
15	Kucukceran	2022	****	*	**	7*
16	Akboga	2017	****	**	***	9*
17	Ding	2023	****	*	**	7*
18	Gencdal	2024	****	*	**	7*

Selection: *:Meet the one itemin the NOS selection section. **:Meet the two items in the NOS selection section. ***: Meet the three items in the NOS selection section. ****:Meet the four items in the NOS selection section.

Comparability: *:The comparability of the study cohort design was of medium quality. **:The comparability of the study cohort designwas of high quality.

Outcome: *:Meet the one itemin the NOS outcome section. **:Meet the two items in the NOS outcome section. ***:Meet the three items in the NOS outcome section.

### Traditional pairwise meta-analysis

The following inflammatory markers were significantly elevated in CVT patients compared with control group (Table 4): WBC (MD = 1.46, 95%CI: 0.46–2.45), neutrophil (MD = 1.78, 95%CI: 0.86–2.7), hs-CRP (MD = 10.92, 95%CI: 6.23–15.61), CRP (MD = 9.18, 95%CI: 1.97–16.38), NLR (MD = 1.48, 95%CI: 0.93–2.04), SII (MD = 403.86, 95%CI: 326.06–481.66), PLR (MD = 31.47, 95%CI: 15.8–47.14), IL-6 (MD = 7.84, 95%CI: 6.41–9.27), monocytes (MD = 0.1, 95%CI: 0.07–0.13) and MHR (MD = 0.09, 95%CI: 0.01–0.17). Conversely, lymphocyte was significantly reduced in CVT patients (MD = −0.31, 95%CI: −0.41, −0.22). A funnel plot evaluating publication bias in the traditional meta-analysis of CVT (Supplementary Figure S1). Forest plots for the traditional meta-analyses of CVT and IS are provided in Supplementary Figure S2.

**Table 4:** Characteristics of the traditional meta-analysis for CVT.

Inflammatory Biomarkers	Number of cases (CVT: healthy controls)	Mean Difference (MD)	95% Confidence Interval (95%CI)	*I^2^* statistic (%)	*p* value
WBC (× 10^9^/L)	444: 668	1.46	0.46, 2.45	90	=0.004
Neutrophil (× 10^9^/L)	484: 708	1.78	0.86, 2.7	91	=0.0001
Lymphocyte (× 10^9^/L)	444: 668	-0.31	−0.41, −0.22	29	<0.00001
Hs-CRP (mg/L)	131: 81	10.92	6.23, 15.61	0	<0.00001
CRP (mg/L)	186: 133	9.18	1.97, 16.38	85	=0.01
NLR	579: 749	1.48	0.93, 2.04	83	<0.00001
SII	236: 153	403.86	326.06, 481.66	2	<0.00001
Monocyte (× 10^9^/L)	312: 233	0.1	0.07, 0.13	35	<0.00001
PLR	392: 430	31.47	15.8, 47.14	73	<0.00001
MHR	126: 100	0.09	0.01, 0.17	57	=0.02
IL-6 (pg/mL)	241: 134	7.84	6.41, 9.27	0	<0.00001

CVT, Cerebral Venous Thrombosis; WBC, White Blood Cell Count; Hs-CRP, High-sensitivity C-reactive Protein; CRP, C-reactive Protein; NLR, Neutrophil-to-Lymphocyte Ratio; SII, Systemic Immune-Inflammation Index; PLR, Platelet-to-Lymphocyte Ratio; MHR, Monocyte-to-High-Density Lipoprotein Cholesterol Ratio; IL-6, Interleukin-6.

Compared with the healthy control group, the following inflammatory markers were significantly elevated in IS patients (Table 5): neutrophil (MD = 1.33, 95%CI: 1.13–1.52), hs-CRP (MD = 3.77, 95%CI: 0.26, 7.28), CRP (MD = 0.98, 95%CI: 0.85–1.11), NLR (MD = 1.51, 95%CI: 0.77–2.25), SII (MD = 326.14, 95%CI: 272.51–379.77), IL-6 (MD = 0.84, 95%CI: 0.55–1.12), MHR (MD = 0.84, 95%CI: 0.59–1.09), WBC (MD = 1.09, 95%CI: 0.81–1.38), monocytes (MD = 0.1, 95%CI: 0.04, 0.16), PLR (MD = 34.8, 95%CI: 8.56, 61.05). Notably, lymphocyte was also reduced in IS patients (MD = −0.60, 95%CI: −0.69, −0.51). A funnel plot evaluating publication bias in the traditional meta-analysis of IS (Supplementary Figure S3).

**Table 5:** Characteristics of the traditional meta-analysis for IS.

Inflammatory Biomarkers	Number of cases (IS: healthy controls)	Mean difference (MD)	95% Confidence Interval (95%CI)	*I^2^* statistic (%)	*p - value*
WBC (× 10^9^/L)	344: 758	1.20	0.63, 1.76	71	<0.00001
Neutrophil (× 10^9^/L)	463: 1112	1.33	1.13, 1.52	18	<0.00001
Lymphocyte (× 10^9^/L)	286: 945	−0.6	−0.69, −0.51	0	<0.00001
Hs-CRP (mg/L)	93: 521	3.77	0.26, 7.28	78	=0.04
CRP (mg/L)	680: 1062	0.98	0.85, 1.11	0	<0.00001
NLR	530: 1109	1.51	0.77, 2.25	94	<0.0001
SII	340: 1001	326.14	272.51, 379.77	0	<0.00001
Monocyte (× 10^9^/L)	606: 1153	0.1	0.04,0.16	89	=0.002
PLR	247: 237	34.8	8.56, 61.05	81	=0.009
MHR	323: 281	0.11	0.07, 0.15	83	<0.00001
IL-6 (pg/mL)	582: 1390	0.84	0.55, 1.12	0	<0.00001

IS, Ischemic stroke; WBC, White Blood Cell Count; Hs-CRP, High-sensitivity C-reactive Protein; CRP, C-reactive Protein; NLR, Neutrophil-to-Lymphocyte Ratio; SII, Systemic Immune-Inflammation Index; PLR, Platelet-to-Lymphocyte Ratio; MHR, Monocyte-to-High-Density Lipoprotein Cholesterol Ratio; IL-6, Interleukin-6.

### Network meta-analysis

CVT showed statistically significant differences in CRP (MD = 7.58, 95% CrI:2.48–14.09) and IL-6 (MD = 6.98, 95% CrI:2.75–11.44) compared with IS. Moreover, the network meta-analysis of CRP and IL-6 in the CVT group also showed significant differences compared with the healthy control group (MD = 8.71, 95% CrI:4.55–14.11), IL-6 (MD = 7.88, 95% CrI:4.86–11.22), suggesting that these two inflammatory biomarkers can serve as key inflammatory markers to distinguish between them. In contrast, comparisons of WBC (MD = −0.23, 95% CrI:−1.66, 1.15), Neutrophil (MD = −0.51, 95% CrI:−2.14, 1.01), Lymphocyte (MD = −0.26, 95% CrI:-0.52, 0.10), hs-CRP (MD = −7.18, 95% CrI:−17.09, 3.40), NLR (MD = −0.12, 95% CrI:−1.82, 1.33), SII (MD = −81.64, 95% CrI:−430.69, 243.60), Monocytes (MD = 0.0005, 95% CrI:−0.13, 0.15), PLR (MD = 2.95, 95% CrI:−32.56, 40.87), MHR (MD = 0.02, 95% CrI:−0.12, 0.17) between IS and CVT patients did not reject the null hypothesis (Table 6). Full network geometry and Forest plots are provided in the Supplementary Figures S4, S5. The main text reports key NMA contrasts with corresponding credible intervals.

**Table 6:** The network meta-analysis for pair-comparisons of inflammatory biomarkers among the IS, CVT and controls.

WBC		
Control		
−1.20 (−2.20, −0.22)	IS	
−1.44 (−2.46, −0.45)	−0.23 (−1.66, 1.15)	CVT

Neutrophil		
Control		
−1.24 (−2.47, 0.01)	IS	
−1.76 (−2.76, −0.82)	−0.51 (−2.14, 1.01)	CVT

Lymphocyte		
Control		
0.59 (0.31, 0.83)	IS	
0.33 (0.19, 0.53)	−0.26 (−0.52, 0.10)	CVT

Hs-CRP		
Control		
−3.69 (−10.07, 1.80)	IS	
−10.95 (−19.27, −2.55)	−7.18 (−17.09, 3.40)	CVT

CRP		
Control		
−1.07 (−4.53, 2.19)	IS	
−8.71 (−14.11, −4.55)	−7.58 (−14.09, −2.48)	CVT

NLR		
Control		
−1.52 (−2.78, −0.29)	IS	
−1.65 (−2.75, −0.81)	−0.12 (−1.82, 1.33)	CVT

SII		
Control		
−331.06 (−575.49, −98.45)	IS	
−413.59 (−669.93, −188.89)	−81.64 (−430.69, 243.60)	CVT

Monocyte		
Control		
−0.10 (−0.21, −0.001)	IS	
−0.10 (−0.19, −0.001)	0.0005 (−0.13, 0.15)	CVT

PLR		
Control		
−34.19 (−63.95, −5.91)	IS	
−31.25 (−53.23, −8.31)	2.95 (−32.56, 40.87)	CVT

MHR		
Control		
−0.11 (−0.21, −0.01)	IS	
−0.09 (−0.20, 0.02)	0.02 (−0.12, 0.17)	CVT

IL-6		
Control		
−0.88 (−3.90, 2.04)	IS	
−7.88 (−11.22, −4.86)	−6.98 (−11.44, −2.75)	CVT

CVT, Cerebral Venous Thrombosis; IS, Ischemic stroke; WBC, White Blood Cell Count; Hs-CRP, High-sensitivity C-reactive Protein; CRP, C-reactive Protein; NLR, Neutrophil-to-Lymphocyte Ratio; SII, Systemic Immune-Inflammation Index; PLR, Platelet-to-Lymphocyte Ratio; MHR, Monocyte-to-High-Density Lipoprotein Cholesterol Ratio; IL-6, Interleukin-6.

### Convergence and risk of bias

The results from the convergence tests (Supplementary Figures S7, S8) revealed that the trace plots of all the parameters stabilized without significant fluctuations in the later iterations, and the density plots exhibited a unimodal and tightly clustered distribution. The shrinkage factor approaches 1 in the final iterations, indicating good model convergence and reliable stability of the results. The funnel plots for the network meta-analysis (Supplementary Figure S6) showed symmetric patterns, and Egger’s regression test detected no significant publication bias (*p* > 0.05). Leave-one-study-out sensitivity analyses, conducted via both traditional and network meta-analysis, yielded results consistent with the primary analysis, with no notable changes in pooled effect sizes or confidence intervals.

### Grade

According to the GRADE assessment, the overall certainty of evidence for all inflammatory markers comparing IS vs. CVT was low, primarily downgraded due to imprecision and indirectness (Table 7).

**Table 7:** GRADE quality of evidence profile.

Markers	Comparison	MD (95% CrI)	Certainty (GRADE)	Interpretation
WBC	IS vs. CVT	−0.23 (−1.66, 1.15)	Low	Imprecision + Indirectness
Neutrophil	IS vs. CVT	−0.51 (−2.14, 1.01)	Low	Imprecision + Indirectness
Lymphocyte	IS vs. CVT	−0.26 (−0.52, 0.10)	Low	Imprecision + Indirectness
Hs-CRP	IS vs. CVT	−7.18 (−17.09, 3.40)	Low	Imprecision + Indirectness
CRP	IS vs. CVT	−7.58 (−14.09, −2.48)	Low	Imprecision + Indirectness
NLR	IS vs. CVT	−0.12 (−1.82, 1.33)	Low	Imprecision + Indirectness
SII	IS vs. CVT	−81.64 (−430.69, 243.60)	Low	Imprecision + Indirectness
Monocyte	IS vs. CVT	0.0005 (−0.13, 0.15)	Low	Imprecision + Indirectness
PLR	IS vs. CVT	2.95 (−32.56, 40.87)	Low	Imprecision + Indirectness
MHR	IS vs. CVT	0.02 (−0.12, 0.17)	Low	Imprecision + Indirectness
IL-6	IS vs. CVT	−6.98 (−11.44, −2.75)	Low	Imprecision + Indirectness

CVT, Cerebral Venous Thrombosis; IS, Ischemic stroke; WBC, White Blood Cell Count; Hs-CRP, High-sensitivity C-reactive Protein; CRP, C-reactive Protein; NLR, Neutrophil-to-Lymphocyte Ratio; SII, Systemic Immune-Inflammation Index; PLR, Platelet-to-Lymphocyte Ratio; MHR, Monocyte-to-High-Density Lipoprotein Cholesterol Ratio; IL-6, Interleukin-6. Effect estimates are presented as mean differences (MD) with 95% credible intervals (CrI) derived from Bayesian network meta-analysis. Certainty of evidence was assessed using the GRADE approach for network meta-analyses. All comparisons between IS and CVT were indirect via the Control node.

### Certainty of evidence

The overall quality of evidence for the pooled results was rated as low, which was attributed primarily to inter-study bias risk, imprecise data, and inconsistent network comparisons. Inter-study bias stemmed from the significant influence of high-risk-of-bias studies on network estimates, which was particularly evident in indirect comparisons between CVT and IS. Imprecision arises from due to insufficient data to demonstrate conclusive effects, whereas inconsistency is caused by a lack of closed loops in the network structure, severely challenging the coherence of comparisons.

## Discussion

Currently, the exploration of the associations between inflammatory biomarkers and CVD continues to deepen. This study included 18 studies and aimed to explore the differences in inflammatory biomarkers between CVT and IS through a systematic review and network meta-analysis. This study transcended the limitations of traditional single-disease research. To our knowledge, this is the first NMA to compare the inflammatory markers of CVT and IS to reveal their common features and specificity in the “inflammation-thrombosis” interactions. However, the inevitable heterogeneity, unclear methodologies, and data transformations require cautious interpretation.

Our analysis revealed significantly elevated levels of IL-6, CRP, hs-CRP, SII, and PLR in the peripheral blood of CVT patients during acute phase. These findings indicate two key pathophysiological processes: (1) a robust systemic inflammatory response, and (2) amplified cross-talk between inflammation and coagulation cascades. This finding is in great agreement with earlier studies: the levels of hs-CRP and IL-6 in the acute stage of CVT are much higher than those in the chronic stage; SII is the strongest independent predictor for CVT, and its ability to predict CVT is better than NLR and PLR (25, 26). The results of this study further support the value of SII in evaluating the thrombo-inflammatory microenvironment. Therefore, for patients with unexplained headache, MRI/MRV is recommended as a priority to screen for CVT when SII is too high (26). As a pro-inflammatory cytokine, IL-6 induces hepatic synthesis of CRP and hs-CRP via the JAK–STAT pathway (33). These acute-phase proteins further to promote tissue factor expression and initiate the extrinsic coagulation pathway to facilitate thrombus formation (18). The significant elevation of SII and PLR reflects neutrophil and platelet overactivation and lymphocyte immunosuppression in the patients. Neutrophils activate endothelial cells and platelets by releasing neutrophil extracellular traps (NETs), reactive oxygen species (ROS), and proinflammatory factors (e.g., IL-6 and TNF-α) to promote coagulation (34, 35). Platelets are not only coagulation elements but also an important sources of inflammatory mediators and a key hubs linking the inflammatory response to thrombosis (5). An increased PLR is independently associated with the risk of recurrent venous thrombosis through a mechanism involving platelet-activated TF expression and increased fibrin production (36). In contrast, although monocytes and MHR showed statistical significance (*p* < 0.05), their effect sizes (MD = 0.1 and 0.09) were lower than that those of IL-6 and CRP. These findings suggest that monocyte-related inflammation may serve as a secondary driver in CVT. However, owing to the statistical significance of the sample size, its clinical value needs to be verified in a larger cohort.

In IS patients, elevated neutrophil levels signify their recruitment to arterial plaque rupture sites. At these sites, neutrophils release myeloperoxidase (MPO) and matrix metalloproteinases (MMPs), which exacerbate endothelial damage and degrade the collagen matrix of the plaque fibrous cap. This process leads to plaque structural instability and promotes ischemic reperfusion injury (37). This process leads to plaque structural instability and promotes ischaemia-reperfusion injury. Hs-CRP is strongly associated with plaque vulnerability, and its mild to moderate elevation indicates chronic inflammation within the plaque (38). Increases in SII and PLR signify synergistic neutrophil–platelet activation: SII indicates an immunocoagulation interaction in thrombus formation, and PLR denotes platelet hyperreactivity, both of which are involved in thromboembolism after plaque rupture (39). An elevated MHR highlights the important role of dysregulated lipid metabolism-inflammation axis interactions in driving atherosclerotic plaque instability. Nonetheless, the analysis of MHR is also limited by sample size, and IS subtype stratification (e.g., large-artery atherosclerosis, cardioembolic stroke, and small-vessel disease) was not performed. This methodological gap likely contributed to heterogeneous inflammatory marker profiles. Future investigations should combine advanced imaging (e.g., plaque component analysis) and molecular approaches (e.g., single-cell RNA sequencing) to dissect subtype-dependent inflammatory pathways, informing the development of targeted antithrombotic and anti-inflammatory strategies.

Compared with the largely focal arterial injury in IS, CVT induces venous congestion-driven endothelial hypoxia and diffuse BBB perturbation, amplifying systemic IL-6/CRP signals. Our network meta-analysis showed that CRP and IL-6 levels are significantly higher in CVT than in IS. This finding highlights a key difference in inflammatory pathways: in CVT, venous endothelial injury triggers a strong systemic acute-phase response, leading to marked elevations in CRP and IL-6. Several studies have shown that serum IL-6 and CRP levels are significantly higher in CVT patients than in healthy controls and are correlated with disease severity (40). Pathophysiologically, venous thrombosis induces endothelial hypoxia and venous congestion, which together provoke a robust systemic acute-phase response. Venous congestion due to impaired venous drainage further exacerbates endothelial injury, leading to a more pronounced local inflammatory response. Endothelial hypoxia in the setting of impaired venous drainage stimulates local IL-6 production and endothelial activation; IL-6 subsequently activates hepatic STAT3 signaling, markedly increasing CRP synthesis and creating an injury-cytokine → acute-phase protein cascade that also upregulates TNF-α and IL-1β (6, 33, 41). In addition, venous stasis and endothelial activation promote neutrophil recruitment and the formation of neutrophil extracellular traps (NETs), a thrombo-inflammatory process that amplifies both thrombus growth and local cytokine release and may further increase systemic IL-6 levels (34, 39). Venous congestion in CVT may produce a relatively more diffuse pattern of blood–brain barrier (BBB) perturbation than the typically focal BBB disruption seen in many cases of arterial ischemia (42); such diffuse BBB disturbance could facilitate translocation of brain-derived cytokines and inflammatory mediators into the systemic circulation and thereby magnify peripheral IL-6 and CRP signals in some patients. In IS, IL-6 is primarily secreted by activated microglia, astrocytes, and cells within arterial plaques in ischemic brain tissue (43). However, the selective permeability of the BBB restricts the diffusion of cytokines such as IL-6 into the systemic circulation, resulting in a significantly less pronounced increase in systemic IL-6 concentrations in IS patients compared to those with CVT (40, 43). This divergence stems from a fundamental difference in injury mechanisms. In CVT, venous thrombosis causes systemic endothelial hypoxia through impaired venous drainage. Conversely, IS results from localized arterial damage due to plaque rupture in cerebral arteries. These distinct pathological processes establish contrasting inflammatory profiles—CVT elicits a strong systemic acute-phase response, whereas IS is characterized by local inflammatory propagation. In IS patients, the mild elevation of CRP and IL-6, likely reflects a postischemic dynamic balance between central and peripheral inflammation: the activation of microglia in the brain results in the release of cytokines, including IL-6, which mediate the dual regulation of neurorestoration and inflammatory injury; concurrently, peripheral monocyte migration into the ischemic penumbra intensifies local oxidative stress, facilitating neuroimmune crosstalk (44). Although this chronic inflammatory state fails to initiate a strong systemic acute-phase response, sustained elevation of CRP is associated with plaque progression and long-term recurrence risk. This provides evidence for statin therapy to improve prognosis through regulating lipid metabolism and exerting anti-inflammatory effects (45). This pathophysiology explains why CRP/IL-6—but not NLR/PLR/SII—best discriminate CVT from IS in our analysis. Given the central role of IL-6 in driving thrombus formation, anti-IL-6 therapies may provide clinical benefits by blocking the IL-6 receptor to reduce inflammation and lower thrombotic risk (46). Recent CVT-specific clinical data show that short-term corticosteroid regimens combined with anticoagulation significantly reduce serum and CSF IL-6 and hs-CRP and are associated with improved functional outcomes, providing indirect clinical support for IL-6–mediated pathology in CVT (47). However, no IL-6–targeted agents have yet been evaluated in patients with CVT, therefore, future prospective randomized trials are warranted to determine the safety, optimal timing, and patient selection for potential anti-IL-6 or other anti-inflammatory interventions.

No significant intergroup differences were observed in other inflammatory markers, likely reflecting stress-induced inflammation inherent to CVD rather than thrombosis-type specific changes. This finding implies a shared core mechanism of systemic inflammation activation across arterial and venous thrombotic disorders. For example, neutrophils are associated with chemokine-mediated recruitment following venous endothelial injury in CVT, whereas in IS, they arise from ischaemic reperfusion injury. Despite distinct pathogenic pathways, neutrophils exhibit similar elevations in both diseases. The levels of novel inflammatory biomarkers, such as NLR, PLR, and SII are markedly increased in both CVT and IS, demonstrating their potential as markers to distinguish healthy individuals from CVD patients. High SII values can independently predict poor prognosis and monitor therapeutic efficacy in these patients (28, 41). These markers (SII, NLR and PLR) are recognized as critical indicators of cerebral arterial ischemia, potentially offering more accurate thrombosis diagnoses than immune cell counts alone (48). Our NMA findings further revealed that these novel inflammatory markers remain prominently elevated in affected patients. Importantly, neuroimaging remains the diagnostic gold standard for both IS and CVT, and the biomarkers identified in our analysis should be regarded as exploratory adjuncts that may complement but cannot substitute for imaging.

The inflammatory signatures of CVT and IS demonstrate both convergence and divergence. Shared mechanisms include activation of neutrophil–platelet crosstalk and systemic immunocoagulative responses, reflected by elevated SII, NLR, and PLR in both conditions. In contrast, CVT exhibits a more pronounced systemic acute-phase reaction, dominated by IL-6–driven CRP elevation and diffuse endothelial activation secondary to venous congestion. Conversely, IS is characterized by localized arterial inflammation, in which monocyte–macrophage and lipid-metabolism–related inflammation, reflected by indices such as MHR and markers linked to plaque vulnerability. Many other markers remain non-specific and overlapping; their elevation likely indexes a shared host response to vascular injury rather than pathognomonic disease-specific biology.

Notably, both CVT and IS groups presented decreased lymphocyte counts. This shared feature may be associated with systemic inflammatory stress responses and immunological homeostasis disruption mediated by vascular injury. Vascular damage under both conditions triggers the innate immune response, leading to the release of proinflammatory cytokines such as IL-6 and TNF-α (49). These cytokines induce lymphocyte apoptosis and promote lymphocyte migration to thrombotic or ischemic foci, leading to a decrease in the number of circulating lymphocytes. IS is associated with more profound lymphopenia. This may be because arterial thrombosis-associated inflammation activates the hypothalamic–pituitary–adrenal (HPA) axis to augment cortisol-mediated immunosuppression. HPA axis activation not only directly inhibits B lymphocyte production but also elevates cortisol levels, which further suppresses lymphocyte proliferation and accelerates apoptosis, forming a “stress–immunosuppression” vicious cycle (50). Additionally, in the ischemic penumbra, bursts of oxygen free radicals, excessive microglial activation, and neutrophil infiltration trigger the release of chemokines (e.g., IL-6, TNF-α and CXCL10), facilitating lymphocyte migration into the brain and subsequent depletion (51). Thus, lymphopenia in CVT and IS likely reflects a combination of vascular injury, inflammatory signaling, and neuroendocrine regulation, with mechanistic disparities tied to thrombus etiology, inflammatory magnitude.

Research indicates that reduced peripheral blood lymphocyte counts in IS patients are significantly associated with the severity of neurological deficits and negatively correlated with infarct volume (52). Clinically, lymphopenia in IS has also been linked to worse neurological outcomes and higher rates of post-stroke infections (53, 54). This association may arise from increased lymphocyte apoptosis due to poststroke immunosuppression and impaired adaptive immunity (53, 54). In CVT patients, although the link between lymphocyte reduction and thrombus burden awaits confirmation, early evidence suggests that lymphopenia may signify a more aggressive thrombotic inflammatory phenotype (55). Abnormalities in lymphocyte count and derived indices such as NLR and PLR indicate a systemic “pro-inflammatory-anti-inflammatory” imbalance—for example, an elevated NLR combined with marked lymphocyte reduction suggests a paradoxical state of coexisting intense immune activation and suppression (41). When lymphopenia co-occurs with elevated IL-6 and CRP, the constellation may represent dysregulated immunity that could both exacerbate tissue injury and increase susceptibility to secondary infections such as pneumonia or urinary tract infection. Future cohorts should implement time-locked immune monitoring (including lymphocyte subsets) and capture secondary infections to delineate the clinical impact of immunosuppression following acute cerebrovascular events.

This study has several limitations. First, the limited number of studies included in the analysis (such as MHR and SII) precludes robust conclusions from the network meta-analysis. As a result, paired comparisons yielded imprecise effect size estimates, which should be interpreted as exploratory evidence rather than definitive findings in the current context. Second, although sensitivity analyses were conducted, inherent bias arose from heterogeneous study designs and variable data collection timepoints across the included studies, which may influence our conclusions. Third, conversion of medians to means for skewed data may introduce additional bias. Additionally, NMA is highly dependent on model specifications, which can increase variance in effect estimates, widen confidence intervals, and potentially compromise conclusion reliability. Finally, owing primarily to the insufficient sample size, subgroup analyses based on CVT and IS subtypes were not conducted. This omission led to excessive heterogeneity in some inflammatory markers. Moreover, geographical publication bias across the included studies and inconsistent measurement methods for certain markers also affected the accuracy and generalizability of the results. These constraints indicate that the present results are hypothesis-generating and require prospective, standardized validation before clinical application.

## Conclusion

This study revealed differences and shared characteristics in the inflammatory marker profiles between CVT and IS. CVT is characterized by systemic inflammatory activation marked by elevated IL-6 and CRP, whereas IS involves localized arterial inflammation (monocytes, MHR) linked to atherosclerotic plaque instability. Network meta-analysis further confirmed that CRP and IL-6 can serve as specific biomarkers to distinguish between these two diseases. Markers such as SII, NLR, and PLR (due to their significant elevation) may have potential as exploratory screening indicators across CVD. The observation of lymphopenia in both CVT and IS patients suggests stress-induced immunosuppression, although the specific mechanisms and clinical implications require further exploration. Given the limitations of this study, these findings should be considered hypothesis-generating and require validation in prospective cohorts before clinical translation.

## Data Availability

The original contributions presented in the study are included in the article/Supplementary material, further inquiries can be directed to the corresponding authors.
