# Correction: Inflammatory biomarkers in cerebral venous thrombosis versus ischemic stroke: a network meta-analysis

**DOI:** 10.3389/fneur.2025.1769992

**Published:** 2026-01-12

**Authors:** Xinlong Shen, Ling Chen, Liling Shen, Shuling Chen, Shuwen Mu, Li Chen, Shousen Wang, Ziqi Li

**Affiliations:** 1 Department of Neurosurgery, Dongfang Affiliated Hospital of Xiamen University, School of Medicine, Xiamen University, Xiamen, Fujian, China; 2 Fujian Provincial Clinical Medical Research Center for Minimally Invasive Diagnosis and Treatment of Neurovascular Diseases, Fuzhou, China; 3 Department of Science and Education, 900th Hospital of PLA Joint Logistic Support Force, Fuzhou, Fujian, China; 4 Department of Epidemiology and Health Statistics, School of Public Health, Fujian Medical University, Fuzhou, Fujian, China; 5 Aberdeen Medical School, College of Life Sciences and Medicine, University of Aberdeen, Aberdeen, United Kingdom; 6 Department of Neurosurgery, Fuzhou University Affiliated Provincial Hospital, Fujian, China; 7 Department of Neurosurgery, Fuzong Clinical Medical College of Fujian Medical University, Fuzhou, Fujian, China

**Keywords:** cerebrovascular disorders, ischemic stroke, cerebral venous thrombosis, inflammation, inflammatory marker

In the published article, there was an error in the affiliation for author Shuling Chen. Affiliation “Department of Epidemiology and Health Statistics, School of Public Health, Fujian Medical University, Fuzhou, Fujian, China” was incorrectly listed for this author. The correct affiliation is “Department of Neurosurgery, Fuzong Clinical Medical College of Fujian Medical University, Fuzhou, Fujian, China”.

In addition, in the published article, author Ziqi Li was erroneously assigned the affiliation “Fujian Provincial Clinical Medical Research Center for Minimally Invasive Diagnosis and Treatment of Neurovascular Diseases, Fuzhou, China”. This affiliation has now been removed for this author. The correct affiliation for Ziqi Li is affiliation 7 only.

The original version of this article has been updated.

