# Supplementary material for: Inflammatory biomarkers in cerebral venous thrombosis versus ischemic stroke: a network meta-analysis
Source: Front Neurol. 2025 Nov 26;16:1634369. doi: 10.3389/fneur.2025.1634369 (PMC12689334; doi:10.3389/fneur.2025.1634369)
Supplement: Supplementary file 1 [file Data_Sheet_1.pdf]

Supplemental Material Table of Contents

Table S1. Search strategies and results

Figure S1. Funnel Plots of Inflammatory Markers in CVT

Figure S2. Traditional meta-analysis Forest plot

Figure S3. Funnel Plots of Inflammatory Markers in IS

Figure S4: The network plot of comparisons for inflammatory marker levels among patients with CVT, IS, and controls.

Figure S5: Forest plot of the network meta-analysis.

Figure S6. Funnel Plots of net-meta analysis

Figure S7. Convergence diagnostics of the network meta-analysis: comparison of inflammatory markers between CVT and IS

Figure S8. Trace and density of the network meta-analysis: comparison of inflammatory markers between CVT and IS

Table S1: Search strategies and results.

Database 1: Pubmed
Search date: 2025:2:1
Search Strategy: (("Ischemic Stroke"[Title] OR "Stroke, Ischemic"[Title] OR "Ischaemic Stroke"[Title] OR "Ischaemic Strokes"[Title] OR "Stroke, Ischaemic"[Title] OR "Acute Ischemic Stroke"[Title] OR "Acute Ischemic Strokes"[Title] OR "Ischemic Stroke, Acute"[Title] OR "Cryptogenic Ischemic Stroke"[Title] OR "Cryptogenic Ischemic Strokes"[Title] OR "Ischemic Stroke, Cryptogenic"[Title] OR "Stroke, Cryptogenic Ischemic"[Title] OR "Cryptogenic Embolism Stroke"[Title] OR "Embolism Stroke, Cryptogenic"[Title] OR "Stroke, Cryptogenic Embolism"[Title] OR "Cryptogenic Stroke"[Title] OR "Stroke, Cryptogenic"[Title] OR "Wake - Up Stroke"[Title] OR "Stroke, Wake - Up"[Title] OR "Ischemic Stroke"[MeSH Terms]) AND ("inflammation"[MeSH Terms] OR "inflammat*"[Title] OR "immun*"[Title] OR "neutrophil"[Title] OR "lymphocyte"[Title] OR "monocyte"[Title] OR "platelet"[Title] OR "c reaction protein"[Title] OR "c-reactive protein"[Title] OR "high sensitivity c reaction protein"[Title] OR "high-sensitivity c-reactive protein"[Title] OR "hs crp"[Title] OR "leukocyte"[Title] OR "white blood cell"[Title] OR "NLR"[Title] OR "neutrophil lymphocyte ratio"[Title] OR "PLR"[Title] OR "platelet lymphocyte ratio"[Title] OR "interleukin 6"[Title] OR "IL-6"[Title] OR "systemic immune inflammation index"[Title] OR "SII"[Title] OR "monocyte high density lipoprotein ratio"[Title] OR "MHR"[Title])) AND ("humans"[Filter]) OR (("Intracranial Thrombosis"[MeSH Terms] OR "Intracranial Thromboses"[Title] OR "Thromboses, Intracranial"[Title] OR "Thrombosis, Intracranial"[Title] OR "Thrombus, Intracranial"[Title] OR "Intracranial Thrombus"[Title] OR "Cerebral Thrombosis"[Title] OR "Cerebral Thromboses"[Title] OR "Thromboses, Cerebral"[Title] OR "Thrombosis, Cerebral"[Title] OR "Brain Thrombosis"[Title] OR "Brain Thromboses"[Title] OR "Thromboses, Brain"[Title] OR "Thrombosis, Brain"[Title] OR "Cerebral

Thrombus"[Title] OR "Thrombus, Cerebral"[Title] OR "Brain Thrombus"[Title] OR "Thrombus, Brain"[Title] OR "Cerebral Venous Thrombosis"[Title] OR "cerebral venous thrombosis"[Title] OR "CVT"[Title]) AND ("inflammation"[MeSH Terms] OR "inflammat*"[Title] OR "immun*"[Title] OR "neutrophil"[Title] OR "lymphocyte"[Title] OR "monocyte"[Title] OR "platelet"[Title] OR "c reaction protein"[Title] OR "c-reactive protein"[Title] OR "high sensitivity c reaction protein"[Title] OR "high-sensitivity c-reactive protein"[Title] OR "hs crp"[Title] OR "leukocyte"[Title] OR "white blood cell"[Title] OR "NLR"[Title] OR "neutrophil lymphocyte ratio"[Title] OR "PLR"[Title] OR "platelet lymphocyte ratio"[Title] OR "interleukin 6"[Title] OR "IL-6"[Title] OR "systemic immune inflammation index"[Title] OR "SII"[Title] OR "monocyte high density lipoprotein ratio"[Title] OR "MHR"[Title])) AND ("humans"[Filter])

Database 2: Embase
Search date: 2025:2:1
Search Strategy: ('ischemic stroke'/exp OR 'brain infarction'/exp OR 'cryptogenic stroke'/de OR 'cerebrovascular accident'/de OR ('ischemic stroke':ti,ab,kw OR 'stroke, ischemic':ti,ab,kw OR 'ischaemic stroke':ti,ab,kw OR 'acute ischemic stroke':ti,ab,kw OR 'cryptogenic ischemic stroke':ti,ab,kw OR 'wake-up stroke':ti,ab,kw OR 'cryptogenic embolism stroke':ti,ab,kw)) AND ('inflammation'/exp OR 'immune system'/exp OR (inflammat*:ti,ab,kw OR immun*:ti,ab,kw OR neutrophil:ti,ab,kw OR lymphocyte:ti,ab,kw OR monocyte:ti,ab,kw OR platelet:ti,ab,kw OR 'c reactive protein':ti,ab,kw OR 'c-reactive protein':ti,ab,kw OR 'high sensitivity c reactive protein':ti,ab,kw OR 'high-sensitivity c-reactive protein':ti,ab,kw OR 'hs crp':ti,ab,kw OR leukocyte:ti,ab,kw OR 'white blood cell':ti,ab,kw OR nlr:ti,ab,kw OR 'neutrophil lymphocyte ratio':ti,ab,kw OR plr:ti,ab,kw OR 'platelet lymphocyte ratio':ti,ab,kw OR 'interleukin 6':ti,ab,kw OR 'il-6':ti,ab,kw OR 'systemic immune inflammation

index':ti,ab,kw OR sii:ti,ab,kw OR 'monocyte high density lipoprotein ratio':ti,ab,kw OR mhr:ti,ab,kw)) AND [humans]/lim OR ('intracranial thrombosis'/exp OR 'cerebral thrombosis'/exp OR 'cerebral vein thrombosis'/exp OR ('intracranial thrombus':ti,ab,kw OR 'cerebral thrombosis':ti,ab,kw OR 'brain thrombosis':ti,ab,kw OR 'cerebral venous thrombosis':ti,ab,kw OR cvt:ti,ab,kw)) AND ('inflammation'/exp OR 'immune system'/exp OR (inflammat*:ti,ab,kw OR immun*:ti,ab,kw OR neutrophil:ti,ab,kw OR lymphocyte:ti,ab,kw OR monocyte:ti,ab,kw OR platelet:ti,ab,kw OR 'c reactive protein':ti,ab,kw OR 'c-reactive protein':ti,ab,kw OR 'high sensitivity c reactive protein':ti,ab,kw OR 'high-sensitivity c-reactive protein':ti,ab,kw OR 'hs crp':ti,ab,kw OR leukocyte:ti,ab,kw OR 'white blood cell':ti,ab,kw OR nlr:ti,ab,kw OR 'neutrophil lymphocyte ratio':ti,ab,kw OR plr:ti,ab,kw OR 'platelet lymphocyte ratio':ti,ab,kw OR 'interleukin 6':ti,ab,kw OR 'il-6':ti,ab,kw OR 'systemic immune inflammation index':ti,ab,kw OR sii:ti,ab,kw OR 'monocyte high density lipoprotein ratio':ti,ab,kw OR mhr:ti,ab,kw)) AND [humans]/lim

Database 3: Cochrane Library
Search date: 2025:2:1
Search Strategy:
#1: MeSH descriptor: [Intracranial Thromboses] explode all trees
#2: (Intracranial Thromboses):ti,ab,kw OR (Thromboses, Intracranial):ti,ab,kw OR (Thrombosis, Intracranial):ti,ab,kw OR (Thrombus, Intracranial):ti,ab,kw OR (Intracranial Thrombus):ti,ab,kw OR (Cerebral Thrombosis):ti,ab,kw OR (Cerebral Thromboses):ti,ab,kw OR (Thromboses, Cerebral):ti,ab,kw OR (Thrombosis, Cerebral):ti,ab,kw OR (Brain Thrombosis):ti,ab,kw OR (Brain Thromboses):ti,ab,kw OR (Thromboses, Brain):ti,ab,kw OR (Thrombosis, Brain):ti,ab,kw OR (Cerebral Thrombus):ti,ab,kw OR (Thrombus, Cerebral):ti,ab,kw OR (Brain Thrombus):ti,ab,kw OR (Thrombus, Brain):ti,ab,kw OR (Cerebral Venous Thrombosis):ti,ab,kw OR (cerebral

venous thrombosis):ti,ab,kw OR (CVT):ti,ab,kw
#3: #1 OR #2
#4: MeSH descriptor: [Ischemic Stroke]
#5: (Ischemic Stroke):ti,ab,kw OR (Stroke, Ischemic):ti,ab,kw OR (Ischaemic Stroke):ti,ab,kw OR (Ischaemic Strokes):ti,ab,kw OR (Stroke, Ischaemic):ti,ab,kw OR (Acute Ischemic Stroke):ti,ab,kw OR (Acute Ischemic Strokes):ti,ab,kw OR (Ischemic Stroke, Acute):ti,ab,kw OR (Cryptogenic Ischemic Stroke):ti,ab,kw OR (Cryptogenic Ischemic Strokes):ti,ab,kw OR (Ischemic Stroke, Cryptogenic):ti,ab,kw OR (Stroke, Cryptogenic Ischemic):ti,ab,kw OR (Cryptogenic Embolism Stroke):ti,ab,kw OR (Embolism Stroke, Cryptogenic):ti,ab,kw OR (Stroke, Cryptogenic Embolism):ti,ab,kw OR (Cryptogenic Stroke):ti,ab,kw OR (Stroke, Cryptogenic):ti,ab,kw OR (Wake-Up Stroke):ti,ab,kw OR (Stroke, Wake-Up):ti,ab,kw
#6:#4 OR #5
#7: MeSH descriptor: [Inflammation] explode all trees
#8:(inflammat*):ti,ab,kw OR (immun*):ti,ab,kw OR (neutrophil):ti,ab,kw OR (lymphocyte):ti,ab,kw OR (monocyte):ti,ab,kw OR (c reaction protein):ti,ab,kw OR (c-reaction protein):ti,ab,kw OR (high-sensitivity-c-reaction protein):ti,ab,kw OR (leukocyte):ti,ab,kw OR (NLR):ti,ab,kw OR (neutrophil lymphocyte ratio):ti,ab,kw OR (PLR):ti,ab,kw OR (white blood cell):ti,ab,kw OR (neutrophil lymphocyte ratio):ti,ab,kw OR (high sensitivity c reaction protein):ti,ab,kw OR (platelet lymphocyte ratio):ti,ab,kw OR (interleukin 6):ti,ab,kw OR (IL-6):ti,ab,kw OR (systemic immune inflammation index):ti,ab,kw OR (SII):ti,ab,kw OR (monocyte high density lipoprotein ratio):ti,ab,kw OR(MHR):ti,ab,kw
#9: #7 OR #8
#10： humans as population
#11: #9 AND #10

#12: #3 AND #11
#13: #6 AND #11
#14: #12 OR #13

Database 4: Web of Science
Search date: 2025:2:1
Search Strategy:
#1 TI=(Ischemic Stroke OR Ischaemic Stroke OR Ischaemic Strokes OR Acute Ischemic Stroke OR Acute Ischemic Strokes OR Cryptogenic Ischemic Stroke OR Cryptogenic Ischemic Strokes OR Cryptogenic Embolism Stroke OR Cryptogenic Stroke OR Wake - Up Stroke)
#2 TI=("inflammation" OR "inflammat*" OR "immun*" OR "neutrophil" OR "lymphocyte" OR "monocyte" OR "platelet" OR "leukocyte" OR "white blood cell" OR "c reaction protein" OR "c-reactive protein" OR "high sensitivity c reaction protein" OR "high-sensitivity c-reactive protein" OR "hs crp" OR "hs-crp" OR "interleukin 6" OR "IL-6" OR "IL 6" OR "systemic immune inflammation index" OR "SII" OR "monocyte high density lipoprotein ratio" OR "MHR" OR "neutrophil lymphocyte ratio" OR "NLR" OR "platelet lymphocyte ratio" OR "PLR")
#3 #1 AND #2
#4 TI=(Intracranial Thrombosis OR Intracranial Thromboses OR Intracranial Thrombus OR Cerebral Thrombosis OR Cerebral Thromboses OR Brain Thrombosis OR Brain Thromboses OR Cerebral Thrombus OR Brain Thrombus OR Cerebral Venous Thrombosis OR cerebral venous thrombosis OR CVT)
#5 #2 AND #4
#6 #3 OR #5

Figure S1: Funnel Plots of Inflammatory Markers in CVT.

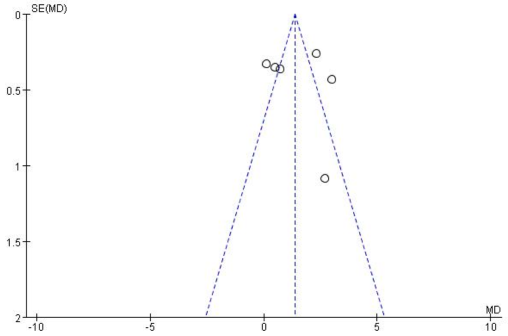

WBC

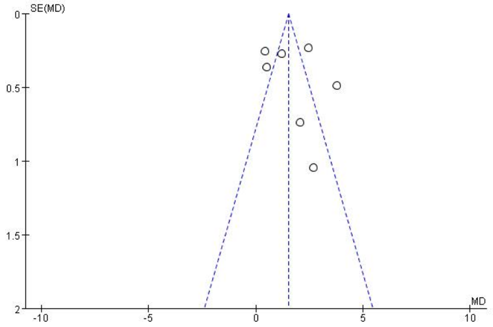

Neutrophil

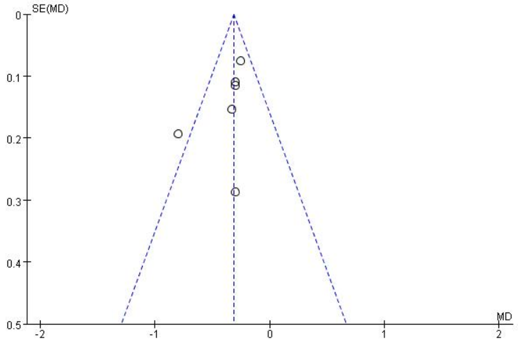

Lymphocyte

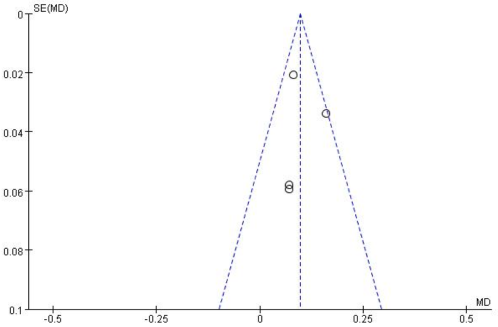

Monocyte

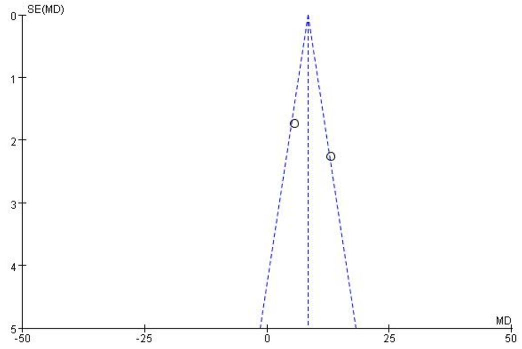

CRP

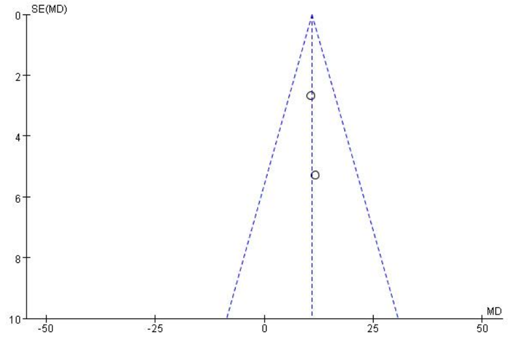

Hs-CRP

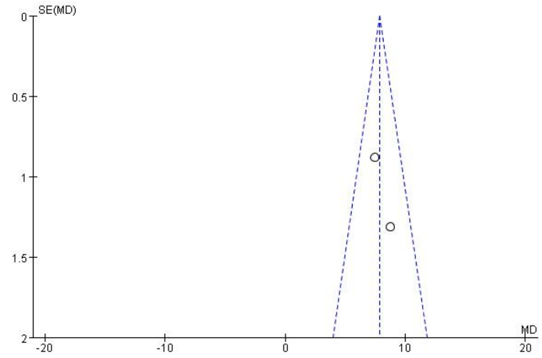

IL-6

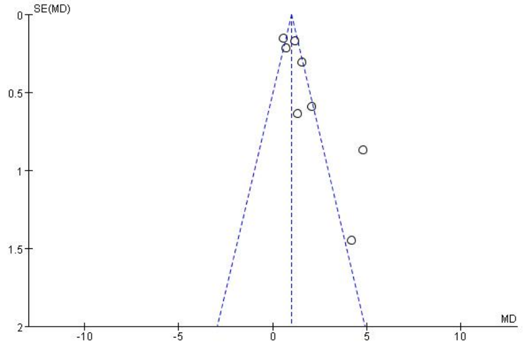

NLR

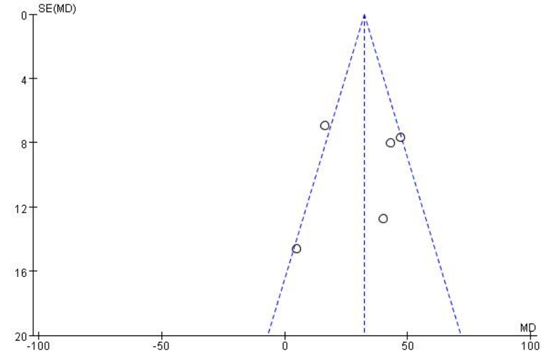

PLR

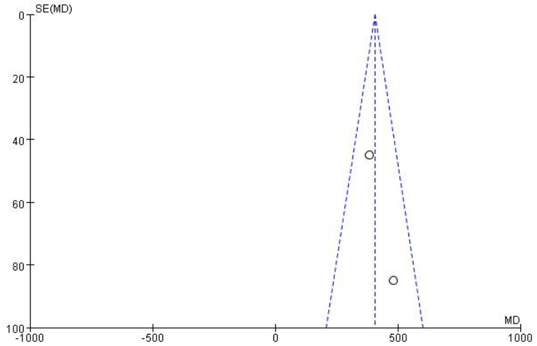

SII

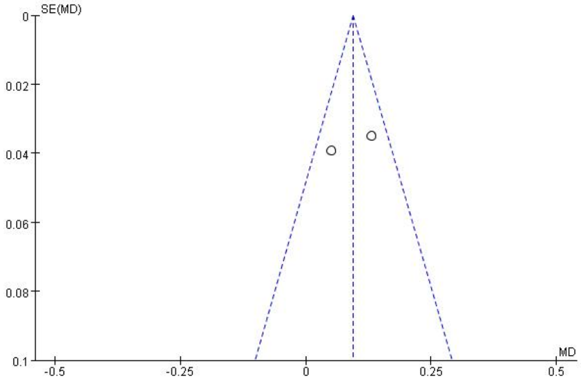

MHR

Figure S1. Publication bias from the included articles about the inflammatory markers of CVT. The graph is symmetrical, which demonstrates that the publication bias of included studies is low.

Figure S2: Traditional meta-analysis Forest plot.

1. WBC

Study or Subgroup	CVT Mean	CVT SD	CVT Total	Control Mean	Control SD	Control Total	Weight	Mean Difference IV, Random, 95% CI
Kamisli, 2012	10.8	5.9	35	8.1	2.2	27	10.3%	2.70 [0.58, 4.82]
Zhang, 2021	9.92	3.42	90	6.93	1.88	60	17.2%	2.99 [2.14, 3.84]
Akboga, 2017	8.4	3.1	80	7.7	1.7	197	17.8%	0.70 [-0.02, 1.42]
Kucukceran, 2022	9.39	2.27	57	8.89	2.94	251	17.9%	0.50 [-0.19, 1.19]
Tekesin, 2019	7.23	1.58	36	7.11	1.27	40	18.1%	0.12 [-0.53, 0.77]
Ding, 2023	7.79	2.69	146	5.5	1.33	93	18.7%	2.29 [1.78, 2.80]
**Total (95% CI)**			**444**			**668**	**100.0%**	**1.46 [0.46, 2.45]**

Heterogeneity: Tau² = 1.31; Chi² = 51.38, df = 5 (P < 0.00001); I² = 90%
Test for overall effect: Z = 2.87 (P = 0.004)

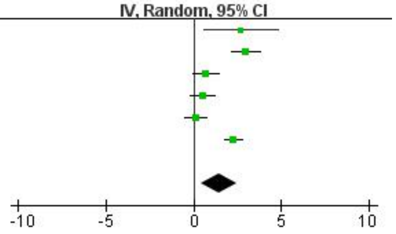

CVT-WBC

Study or Subgroup	CIS Mean	CIS SD	CIS Total	Control Mean	Control SD	Control Total	Weight	Mean Difference IV, Random, 95% CI
Korkut, 2022	9.18	3.31	57	9	2.84	41	11.5%	0.18 [-1.04, 1.40]
Chang, 2005	8.2	2.7	33	6	1.5	41	13.7%	2.20 [1.17, 3.23]
Tekesin, 2023	8.8	2.9	70	6.9	1.8	70	16.6%	1.90 [1.10, 2.70]
Chang, 2005	7.5	1.9	35	6	1.5	41	16.9%	1.50 [0.72, 2.28]
Miwa, 2013	6	1.39	25	5.59	1.45	439	20.0%	0.41 [-0.15, 0.97]
Gencdal, 2024	8.16	2.08	124	7.11	1.71	126	21.2%	1.05 [0.58, 1.52]
**Total (95% CI)**			**344**			**758**	**100.0%**	**1.20 [0.63, 1.76]**

Heterogeneity: Tau² = 0.33; Chi² = 17.26, df = 5 (P = 0.004); I² = 71%
Test for overall effect: Z = 4.14 (P < 0.0001)

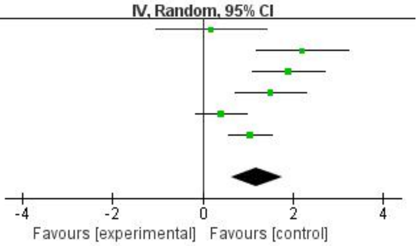

IS-WBC

2. Neutrophil

Study or Subgroup	CVT Mean	CVT SD	CVT Total	Control Mean	Control SD	Control Total	Weight	Mean Difference IV, Random, 95% CI
Akboga, 2017	5.6	2.3	80	4.4	1.4	197	16.2%	1.20 [0.66, 1.74]
Ding, 2023	5.59	2.41	146	3.14	1.18	93	16.4%	2.45 [1.99, 2.91]
Kamisli, 2012	7.8	5.6	35	5.1	2.3	27	9.2%	2.70 [0.65, 4.75]
Kucukceran, 2022	6.85	2.23	57	6.36	3.4	251	15.5%	0.49 [-0.23, 1.21]
kula, 2024	6.72	4.3	40	4.65	1.82	40	12.0%	2.07 [0.62, 3.52]
Tekesin, 2019	4.49	1.23	36	4.08	1	40	16.3%	0.41 [-0.10, 0.92]
Zhang, 2021	7.69	4.38	90	3.91	1.24	60	14.4%	3.78 [2.82, 4.74]
**Total (95% CI)**			**484**			**708**	**100.0%**	**1.78 [0.86, 2.70]**

Heterogeneity: Tau² = 1.28; Chi² = 66.70, df = 6 (P < 0.00001); I² = 91%
Test for overall effect: Z = 3.81 (P = 0.0001)

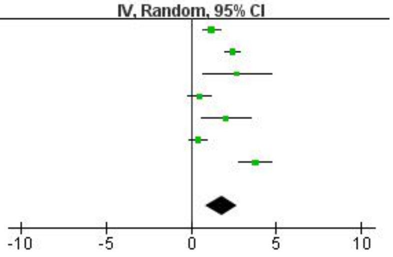

CVT- Neutrophil

Study or Subgroup	CIS Mean	CIS SD	CIS Total	Control Mean	Control SD	Control Total	Weight	Mean Difference IV, Fixed, 95% CI
Gencdal, 2024	5.2	2.18	124	4.13	1.35	126	18.5%	1.07 [0.62, 1.52]
Korkut, 2022	6.41	3.13	53	5.68	2.61	41	2.8%	0.73 [-0.43, 1.89]
Tekesin, 2023	5.7	2.6	70	4	1	70	8.8%	1.70 [1.05, 2.35]
Weng, 2021	4.79	1.64	216	3.42	1.17	875	69.8%	1.37 [1.14, 1.60]
**Total (95% CI)**			**463**			**1112**	**100.0%**	**1.33 [1.13, 1.52]**

Heterogeneity: Chi² = 3.65, df = 3 (P = 0.30); I² = 18%
Test for overall effect: Z = 13.40 (P < 0.00001)

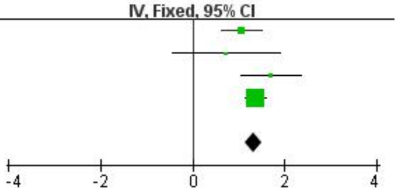

IS- Neutrophil

3. Lymphocyte

Study or Subgroup	CVT Mean	CVT SD	CVT Total	Control Mean	Control SD	Control Total	Weight	Mean Difference IV, Fixed, 95% CI
Akboga, 2017	2.2	0.9	80	2.5	0.8	197	18.2%	-0.30 [-0.53, -0.07]
Ding, 2023	1.73	0.72	146	1.98	0.46	93	41.8%	-0.25 [-0.40, -0.10]
Kamisli, 2012	1.9	1.6	35	2.2	0.5	27	3.0%	-0.30 [-0.86, 0.26]
Kucukceran, 2022	1.7	0.73	57	2	0.84	251	20.0%	-0.30 [-0.52, -0.08]
Tekesin, 2019	1.91	0.46	36	2.71	1.12	40	6.5%	-0.80 [-1.18, -0.42]
Zhang, 2021	1.89	1.19	90	2.22	0.68	60	10.4%	-0.33 [-0.63, -0.03]
**Total (95% CI)**			**444**			**668**	**100.0%**	**-0.31 [-0.41, -0.22]**

Heterogeneity: Chi² = 7.09, df = 5 (P = 0.21); I² = 29%
Test for overall effect: Z = 6.38 (P < 0.00001)

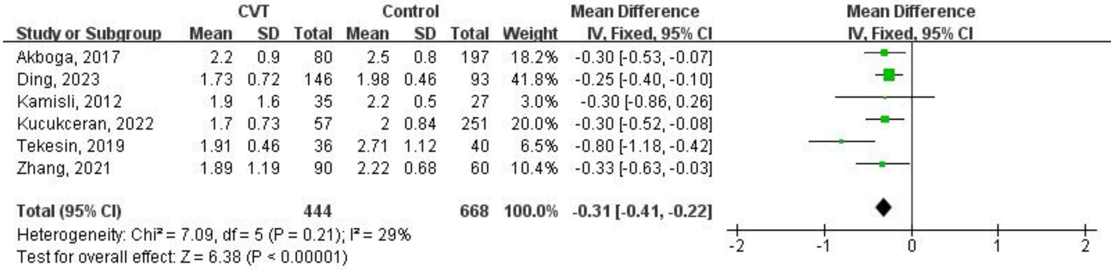

CVT- Lymphocyte

Study or Subgroup	CIS Mean	CIS SD	CIS Total	Control Mean	Control SD	Control Total	Weight	Mean Difference IV, Fixed, 95% CI
Tekesin, 2023	2.1	1.2	70	2.6	0.6	70	8.8%	-0.50 [-0.81, -0.19]
Weng, 2021	1.64	0.67	216	2.25	0.6	875	91.2%	-0.61 [-0.71, -0.51]
**Total (95% CI)**			**286**			**945**	**100.0%**	**-0.60 [-0.69, -0.51]**

Heterogeneity: Chi² = 0.43, df = 1 (P = 0.51); I² = 0%
Test for overall effect: Z = 12.60 (P < 0.00001)

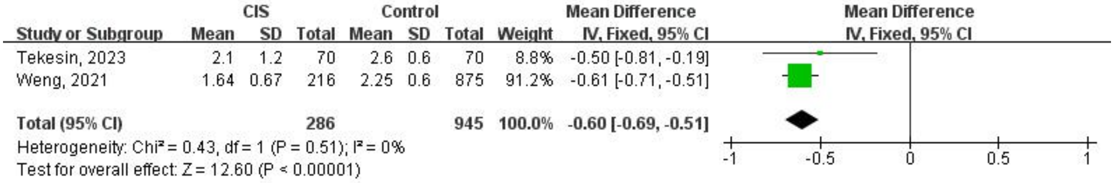

IS- Lymphocyte

4. Monocyte

Study or Subgroup	CVT Mean	CVT SD	CVT Total	Control Mean	Control SD	Control Total	Weight	Mean Difference IV, Fixed, 95% CI
Ding, 2023	0.43	0.21	146	0.35	0.11	93	61.3%	0.08 [0.04, 0.12]
kula, 2024	0.64	0.26	40	0.57	0.27	40	7.5%	0.07 [-0.05, 0.19]
Tekesin, 2019	0.57	0.27	36	0.5	0.23	40	7.9%	0.07 [-0.04, 0.18]
Zhang, 2021	0.65	0.27	90	0.49	0.14	60	23.3%	0.16 [0.09, 0.23]
**Total (95% CI)**			**312**			**233**	**100.0%**	**0.10 [0.07, 0.13]**

Heterogeneity: Chi² = 4.59, df = 3 (P = 0.20); I² = 35%
Test for overall effect: Z = 5.97 (P < 0.00001)

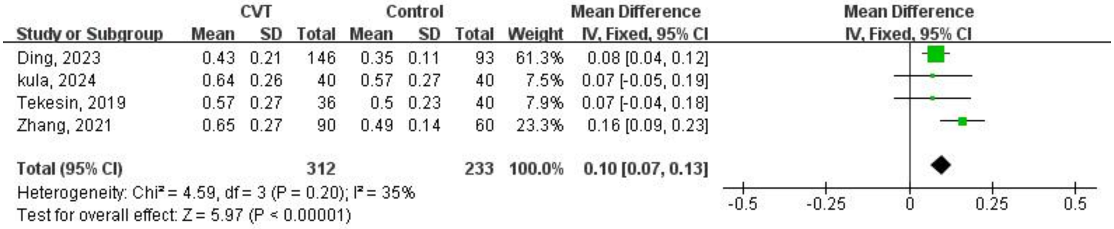

CVT- Monocyte

Study or Subgroup	CIS Mean	CIS SD	CIS Total	Control Mean	Control SD	Control Total	Weight	Mean Difference IV, Random, 95% CI
Gao, 2021	0.48	0.27	283	0.44	0.15	872	35.3%	0.04 [0.01, 0.07]
Liu, 2020	0.39	0.14	253	0.31	0.1	211	37.4%	0.08 [0.06, 0.10]
Tekesin, 2023	0.7	0.2	70	0.5	0.2	70	27.3%	0.20 [0.13, 0.27]
**Total (95% CI)**			**606**			**1153**	**100.0%**	**0.10 [0.04, 0.16]**

Heterogeneity: Tau² = 0.00; Chi² = 18.14, df = 2 (P = 0.0001); I² = 89%
Test for overall effect: Z = 3.08 (P = 0.002)

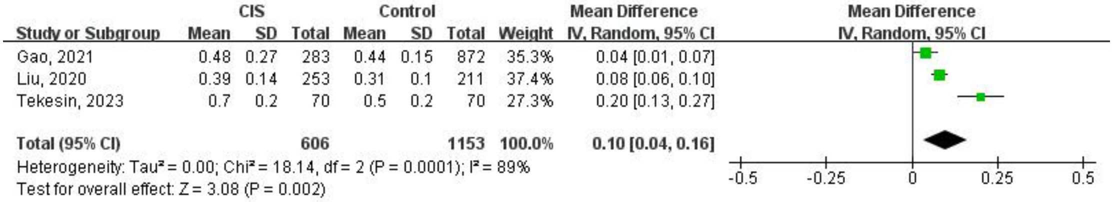

IS- Monocyte

5. PLR

Study or Subgroup	CVT Mean	CVT SD	CVT Total	Control Mean	Control SD	Control Total	Weight	Mean Difference IV, Random, 95% CI
Akboga, 2017	148	61	80	101	50	197	22.7%	47.00 [31.92, 62.08]
Ding, 2023	142.13	58.98	146	125.96	47.42	93	23.7%	16.17 [2.59, 29.75]
kula, 2024	149.25	72.18	40	109.08	35.87	40	16.6%	40.17 [15.19, 65.15]
Tekesin, 2019	113.2	80.19	36	108.28	37.4	40	14.7%	4.92 [-23.72, 33.56]
Zhang, 2021	148.33	64.02	90	105.47	33.77	60	22.3%	42.86 [27.11, 58.61]
**Total (95% CI)**			**392**			**430**	**100.0%**	**31.47 [15.80, 47.14]**

Heterogeneity: Tau² = 222.01; Chi² = 14.69, df = 4 (P = 0.005); I² = 73%
Test for overall effect: Z = 3.94 (P < 0.0001)

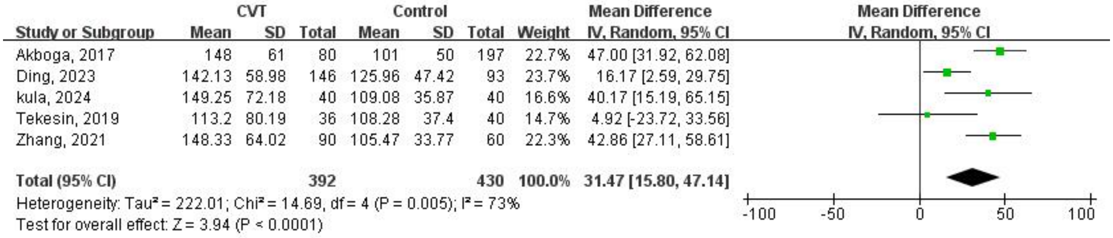

CVT- PLR

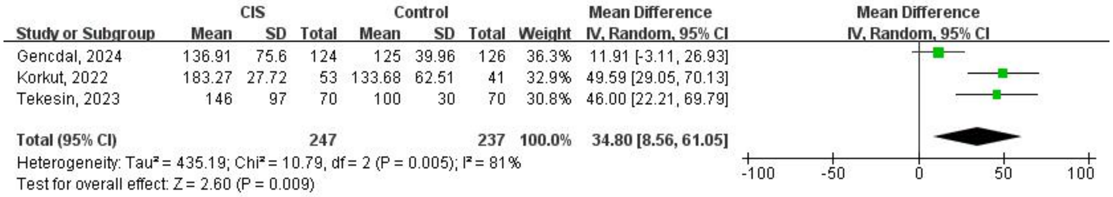

IS- PLR

6. NLR

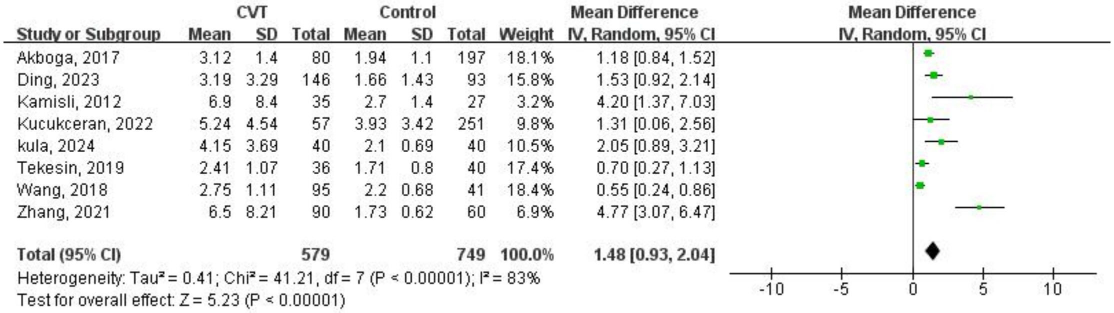

CVT- NLR

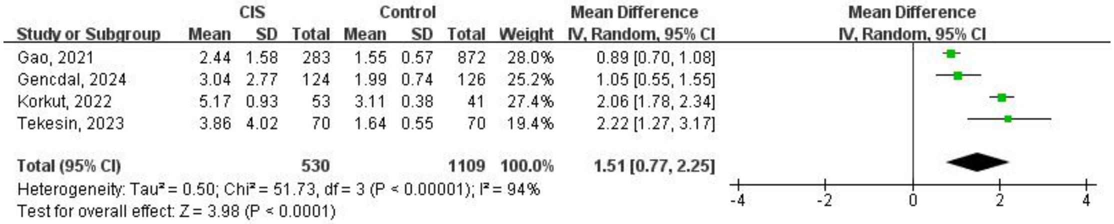

IS- NLR

7. Hs-CRP

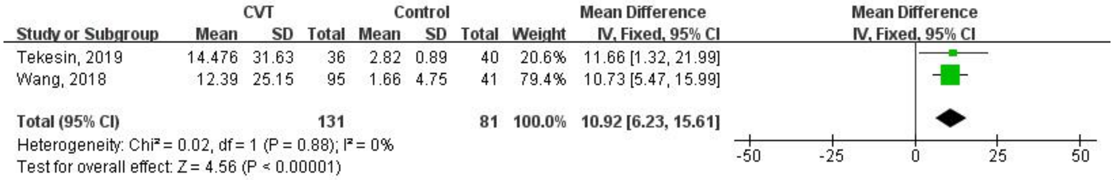

CV

T- Hs-CRP

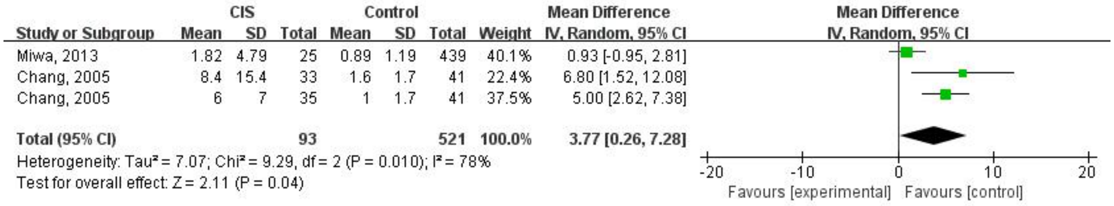

IS- Hs-CRP

8. CRP

Study or Subgroup	CVT Mean	CVT SD	CVT Total	Control Mean	Control SD	Control Total	Weight	Mean Difference IV, Random, 95% CI
Ding, 2023	15.56	27.25	146	2.56	1.39	93	48.1%	13.00 [8.57, 17.43]
kula, 2024	6.31	10.96	40	0.67	0.57	40	51.9%	5.64 [2.24, 9.04]
**Total (95% CI)**			**186**			**133**	**100.0%**	**9.18 [1.97, 16.38]**

Heterogeneity: Tau² = 23.03; Chi² = 6.67, df = 1 (P = 0.010); I² = 85%
Test for overall effect: Z = 2.50 (P = 0.01)

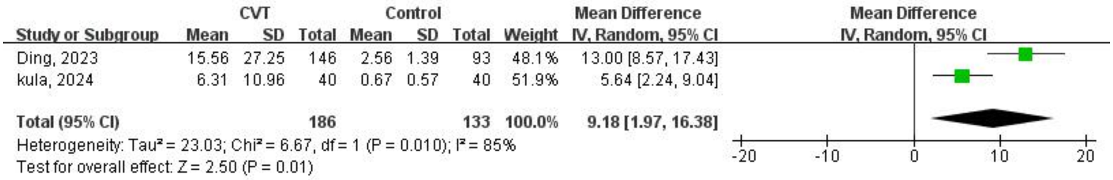

CVT- CRP

Study or Subgroup	CIS Mean	CIS SD	CIS Total	Control Mean	Control SD	Control Total	Weight	Mean Difference IV, Fixed, 95% CI
Jenny, 2019	5.7	8.9	557	4.2	7	951	2.3%	1.50 [0.64, 2.36]
Korkut, 2022	1.57	0.5	53	0.62	0.09	41	88.8%	0.95 [0.81, 1.09]
Tekesin, 2023	1.46	1.84	70	0.34	0.12	70	9.0%	1.12 [0.69, 1.55]
**Total (95% CI)**			**680**			**1062**	**100.0%**	**0.98 [0.85, 1.11]**

Heterogeneity: Chi² = 1.98, df = 2 (P = 0.37); I² = 0%
Test for overall effect: Z = 14.80 (P < 0.00001)

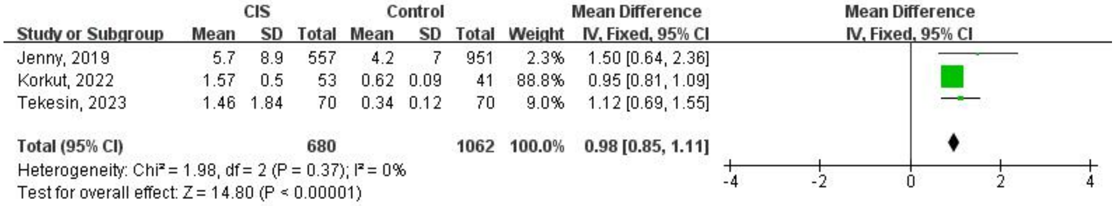

IS- CRP

9. IL-6

Study or Subgroup	CVT Mean	CVT SD	CVT Total	Control Mean	Control SD	Control Total	Weight	Mean Difference IV, Fixed, 95% CI
Ding, 2023	11.91	15.5	146	3.18	2.63	93	31.0%	8.73 [6.16, 11.30]
Wang, 2018	11.53	8.45	95	4.09	0.95	41	69.0%	7.44 [5.72, 9.16]
**Total (95% CI)**			**241**			**134**	**100.0%**	**7.84 [6.41, 9.27]**

Heterogeneity: Chi² = 0.67, df = 1 (P = 0.41); I² = 0%
Test for overall effect: Z = 10.73 (P < 0.00001)

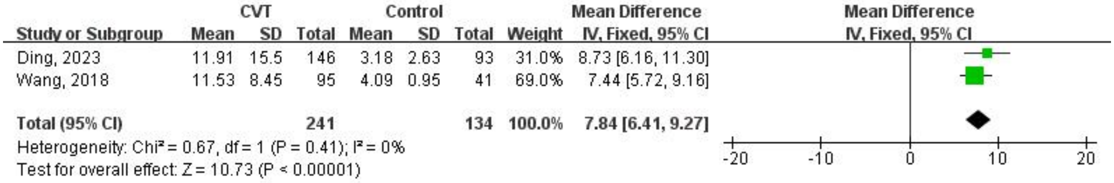

CVT- IL-6

Study or Subgroup	CIS Mean	CIS SD	CIS Total	Control Mean	Control SD	Control Total	Weight	Mean Difference IV, Random, 95% CI
Jenny, 2019	4.5	3.1	557	3.7	2.6	951	87.0%	0.80 [0.49, 1.11]
Miwa, 2013	2.79	1.99	25	1.7	1.39	439	13.0%	1.09 [0.30, 1.88]
**Total (95% CI)**			**582**			**1390**	**100.0%**	**0.84 [0.55, 1.12]**

Heterogeneity: Tau² = 0.00; Chi² = 0.45, df = 1 (P = 0.50); I² = 0%
Test for overall effect: Z = 5.75 (P < 0.00001)

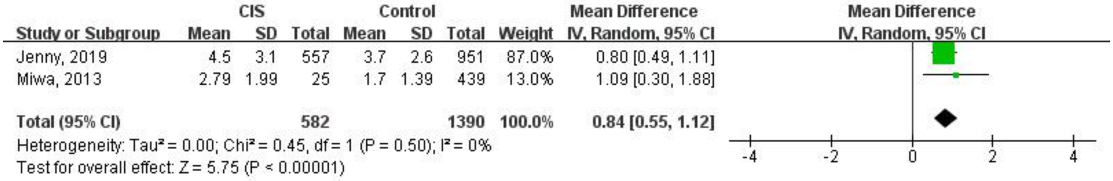

IS- IL-6

10. SII

Study or Subgroup	CVT Mean	CVT SD	CVT Total	Control Mean	Control SD	Control Total	Weight	Mean Difference IV, Fixed, 95% CI
Ding, 2023	798.86	449.05	146	416.16	243.01	93	78.1%	382.70 [294.70, 470.70]
Zhang, 2021	896.84	764.42	90	417.32	207.39	60	21.9%	479.52 [313.10, 645.94]
**Total (95% CI)**			**236**			**153**	**100.0%**	**403.86 [326.06, 481.66]**

Heterogeneity: Chi² = 1.02, df = 1 (P = 0.31); I² = 2%
Test for overall effect: Z = 10.17 (P < 0.00001)

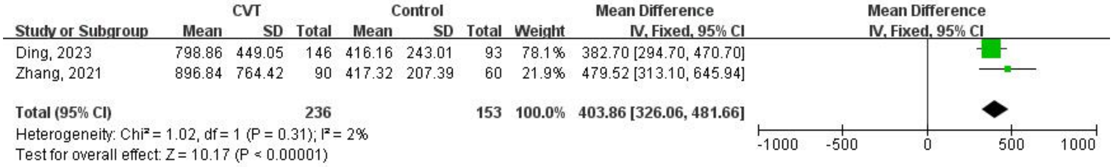

CVT-SII

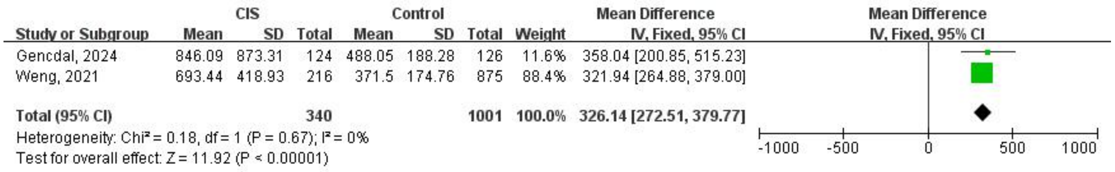

IS-SII

11. MHR

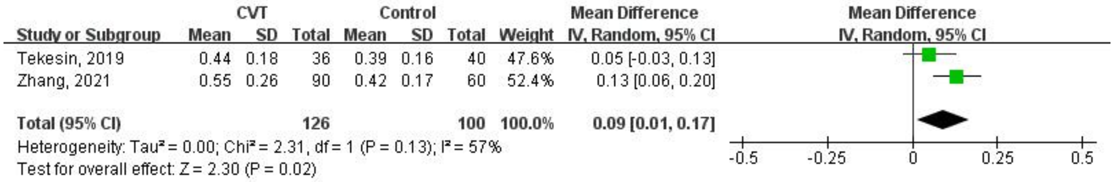

CVT-MHR

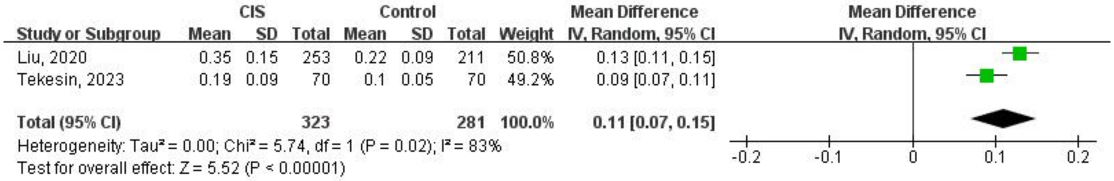

IS-MHR

Abbreviations: CVT, Cerebral Venous Thrombosis; IS, Ischemic Stroke; WBC, White Blood Cell Count; Hs-CRP, High-sensitivity C-reactive Protein; CRP, C-reactive Protein; NLR, Neutrophil-to-Lymphocyte Ratio; SII, Systemic Immune-Inflammation Index; PLR, Platelet-to-Lymphocyte Ratio; MHR, Monocyte-to-High-Density Lipoprotein Cholesterol Ratio; IL-6, Interleukin-6. Mean differences are reported in original study units (Hs-CRP, CRP: mg/L; IL-6: pg/mL); cell counts ($\times 10^9$/L).

Figure S3: Funnel Plots of Inflammatory Markers in IS.

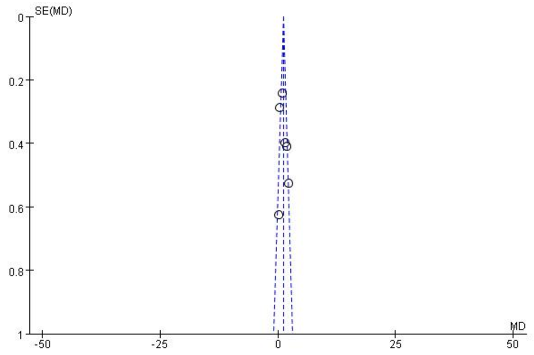

WBC

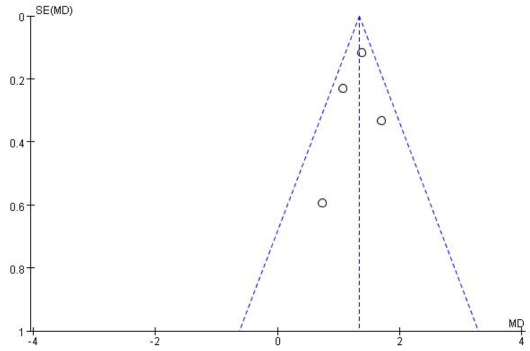

Neutrophil

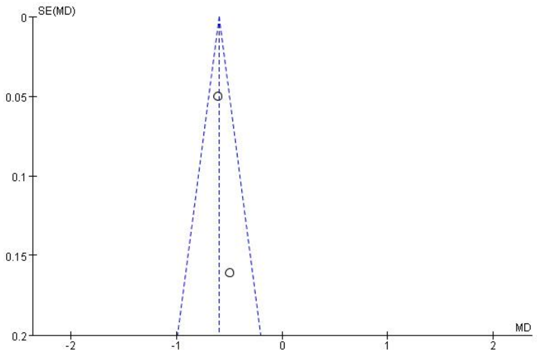

Lymphocyte

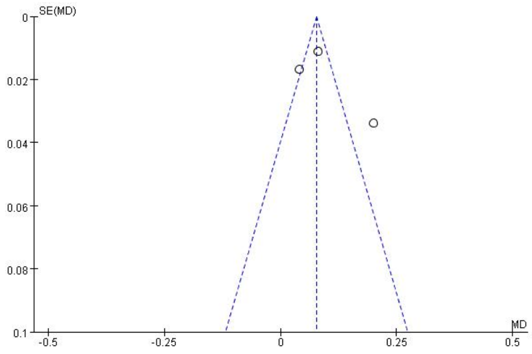

Monocyte

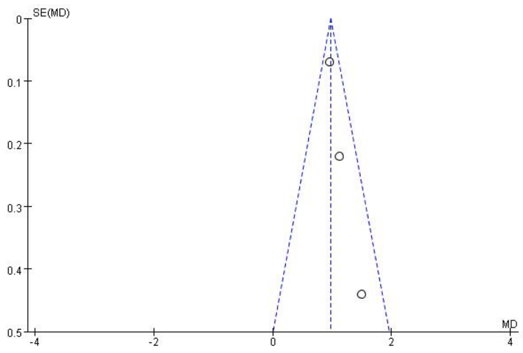

CRP

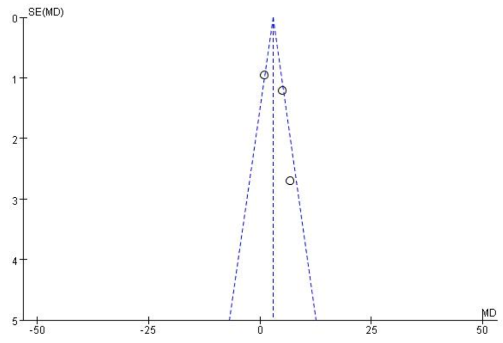

Hs-CRP

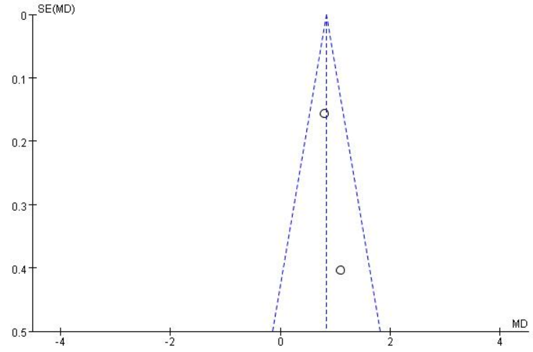

IL-6

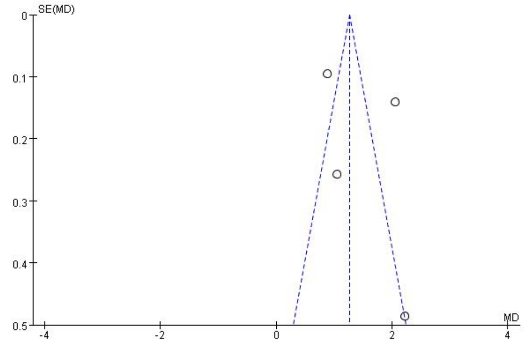

NLR

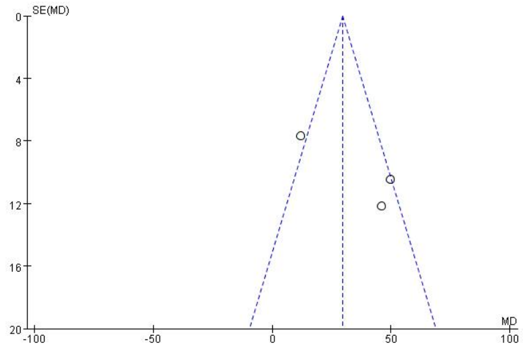

PLR

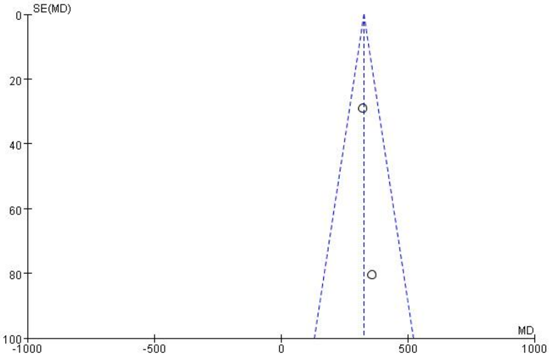

SII

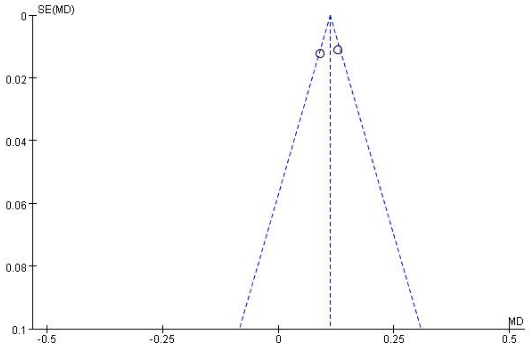

MHR

Figure S3. Publication bias from the included articles about the inflammatory markers of IS. The graph is symmetrical, which demonstrates that the publication bias of included studies is low.

Figure S4: The network plot of comparisons for inflammatory marker levels among patients with CVT, IS, and controls.

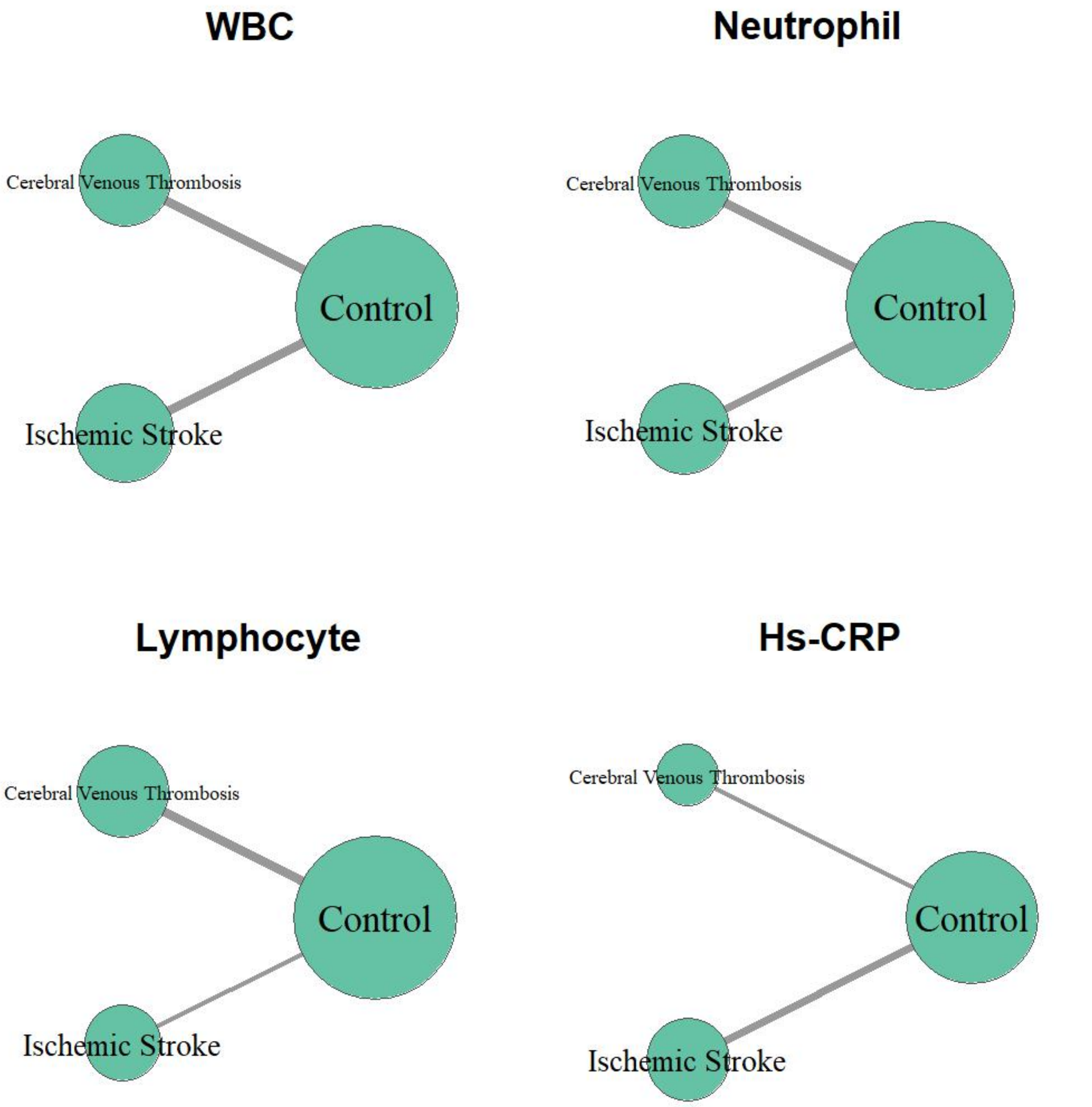

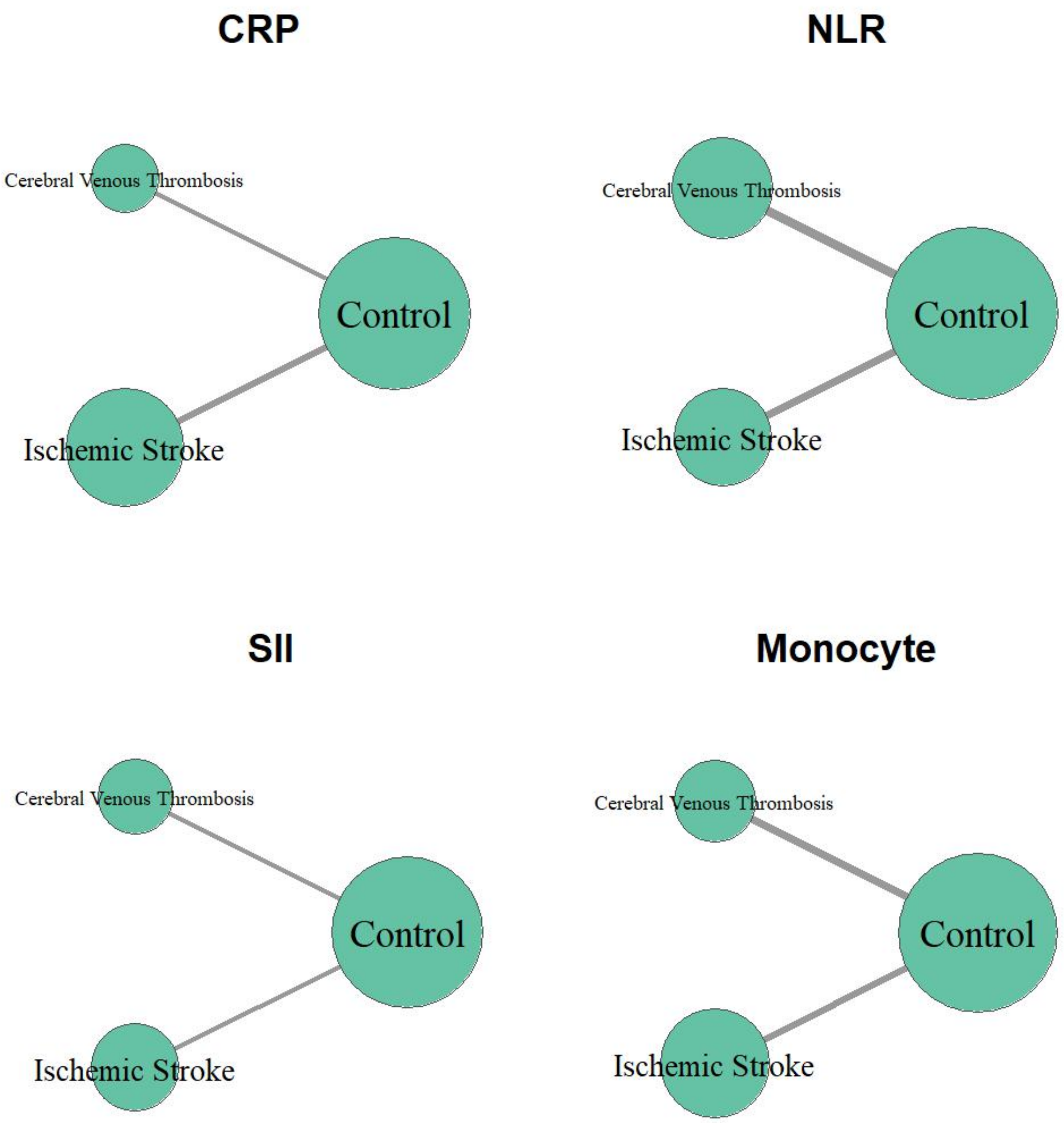
CRP
Cerebral Venous Thrombosis
Control
Ischemic Stroke
NLR
Cerebral Venous Thrombosis
Control
Ischemic Stroke
SII
Cerebral Venous Thrombosis
Control
Ischemic Stroke
Monocyte
Cerebral Venous Thrombosis
Control
Ischemic Stroke

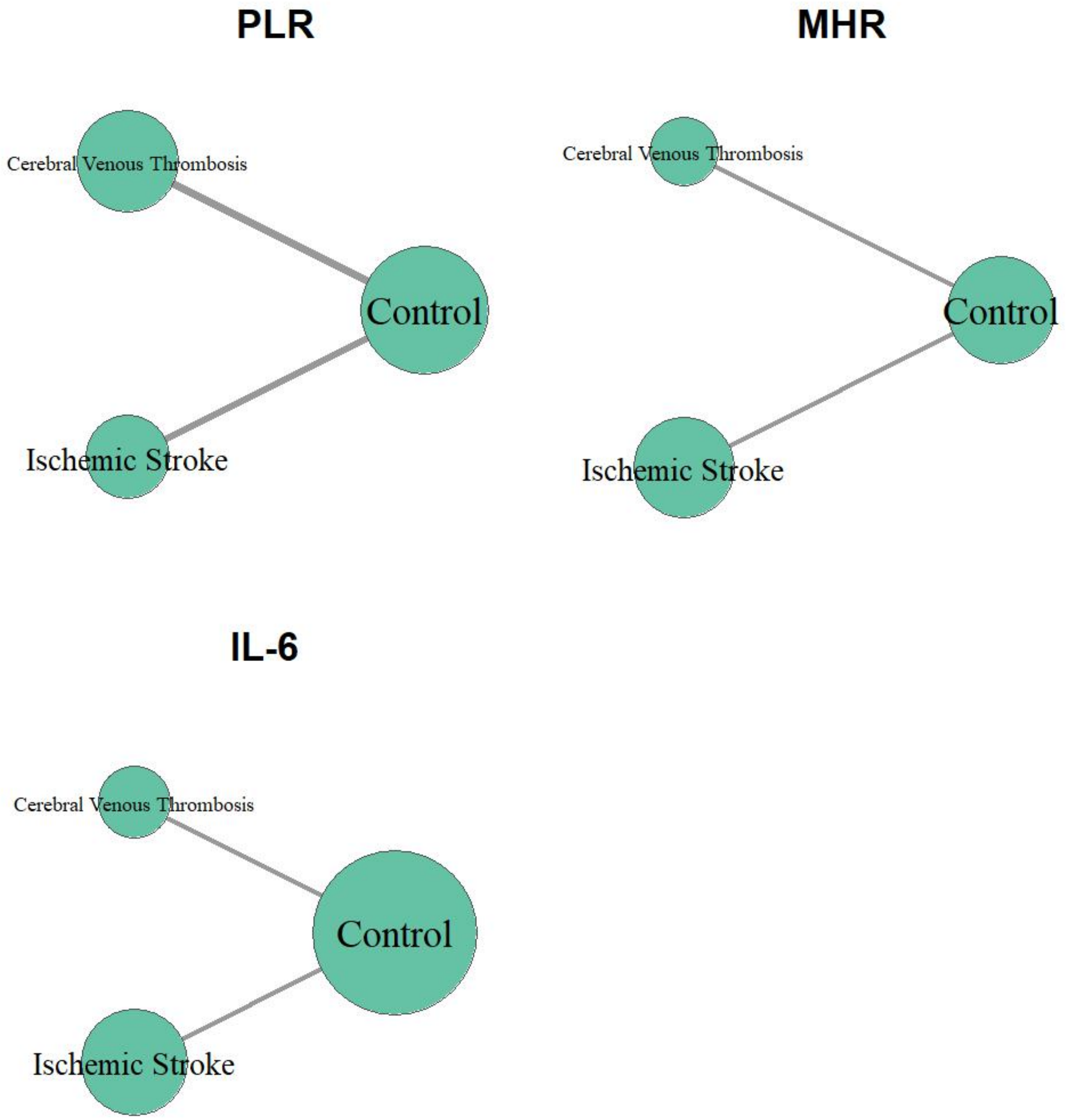

Abbreviations: CVT, Cerebral Venous Thrombosis; IS, Ischemic Stroke; WBC, White Blood Cell Count; Hs-CRP, High-sensitivity C-reactive Protein; CRP, C-reactive Protein; NLR, Neutrophil-to-Lymphocyte Ratio; SII, Systemic Immune-Inflammation Index; PLR, Platelet-to-Lymphocyte Ratio; MHR, Monocyte-to-High-Density Lipoprotein Cholesterol Ratio; IL-6, Interleukin-6. Each node represents a group. The size of each node is proportional to the total sample size of that group. The thickness of the connecting lines reflects the number of studies providing direct comparisons between groups.

Figure S5: Forest plot of the network meta-analysis.

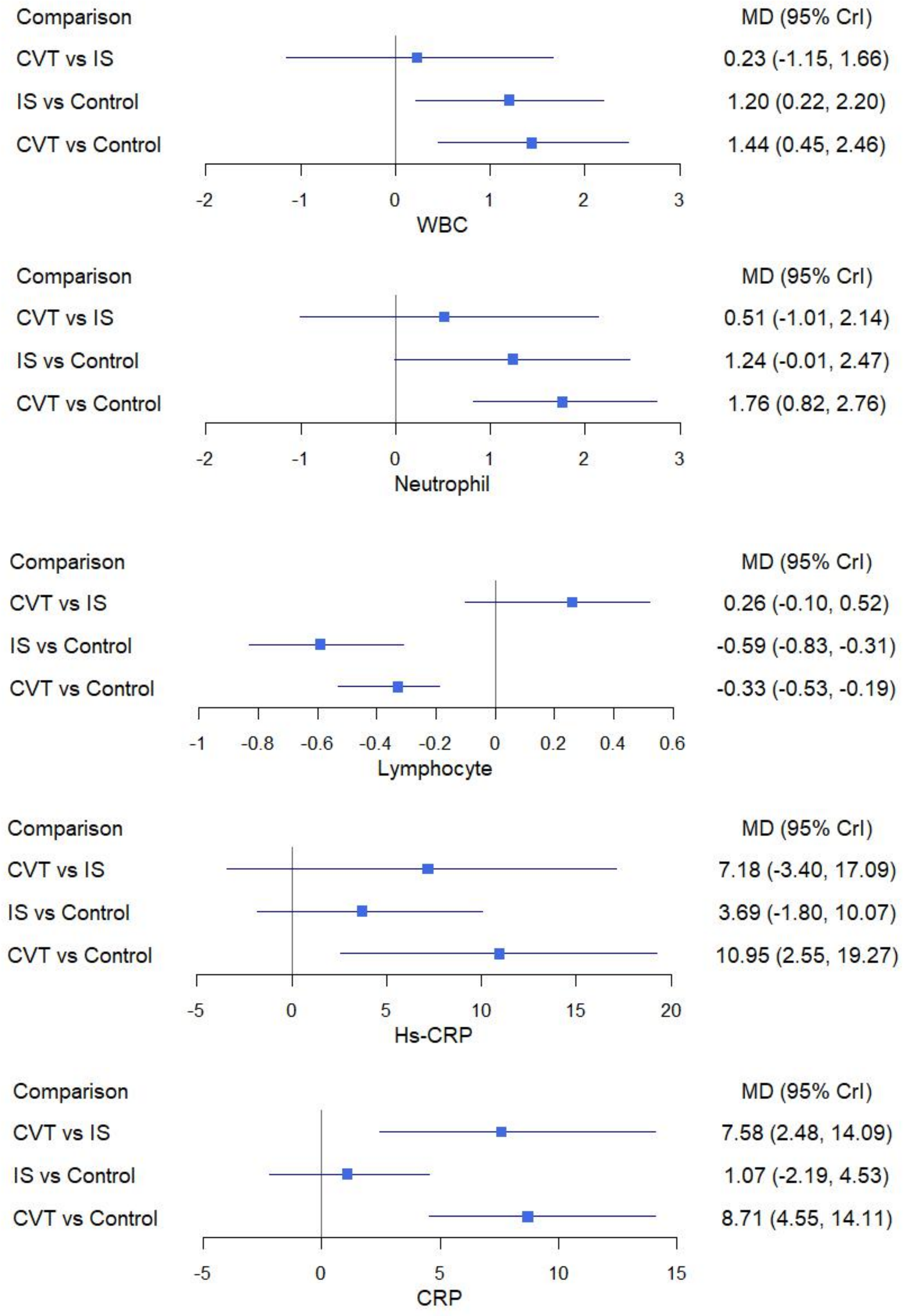

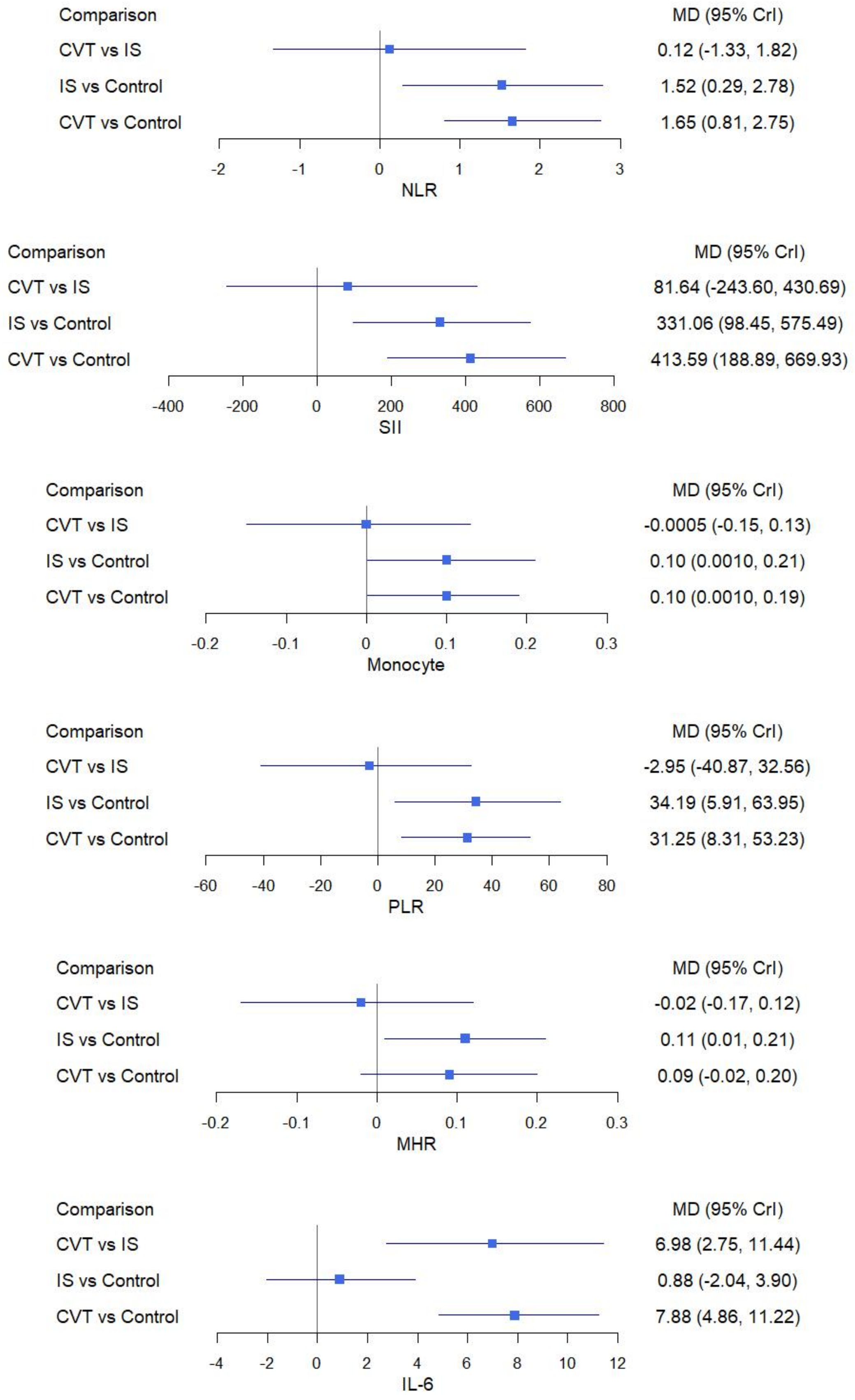
Comparison
MD (95% CrI)
CVT vs IS
0.12 (-1.33, 1.82)
IS vs Control
1.52 (0.29, 2.78)
CVT vs Control
1.65 (0.81, 2.75)
-2 -1 0 1 2 3
NLR
Comparison
MD (95% CrI)
CVT vs IS
81.64 (-243.60, 430.69)
IS vs Control
331.06 (98.45, 575.49)
CVT vs Control
413.59 (188.89, 669.93)
-400 -200 0 200 400 600 800
SII
Comparison
MD (95% CrI)
CVT vs IS
-0.0005 (-0.15, 0.13)
IS vs Control
0.10 (0.0010, 0.21)
CVT vs Control
0.10 (0.0010, 0.19)
-0.2 -0.1 0 0.1 0.2 0.3
Monocyte
Comparison
MD (95% CrI)
CVT vs IS
-2.95 (-40.87, 32.56)
IS vs Control
34.19 (5.91, 63.95)
CVT vs Control
31.25 (8.31, 53.23)
-60 -40 -20 0 20 40 60 80
PLR
Comparison
MD (95% CrI)
CVT vs IS
-0.02 (-0.17, 0.12)
IS vs Control
0.11 (0.01, 0.21)
CVT vs Control
0.09 (-0.02, 0.20)
-0.2 -0.1 0 0.1 0.2 0.3
MHR
Comparison
MD (95% CrI)
CVT vs IS
6.98 (2.75, 11.44)
IS vs Control
0.88 (-2.04, 3.90)
CVT vs Control
7.88 (4.86, 11.22)
-4 -2 0 2 4 6 8 10 12
IL-6

Abbreviations: CVT, Cerebral Venous Thrombosis; IS, Ischemic Stroke; WBC, White Blood Cell Count; Hs-CRP, High-sensitivity C-reactive Protein; CRP, C-reactive Protein; NLR, Neutrophil-to-Lymphocyte Ratio; SII, Systemic Immune-Inflammation Index; PLR, Platelet-to-Lymphocyte Ratio; MHR, Monocyte-to-High-Density Lipoprotein Cholesterol Ratio; IL-6, Interleukin-6.

Figure S6: Funnel Plots of net-meta analysis.

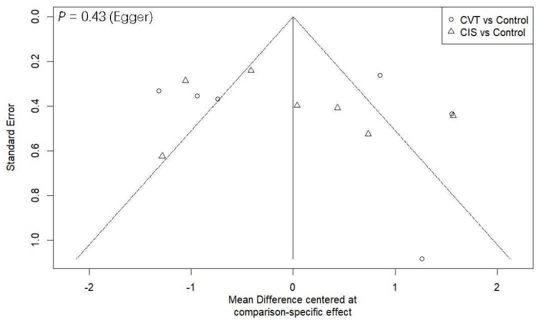

WBC

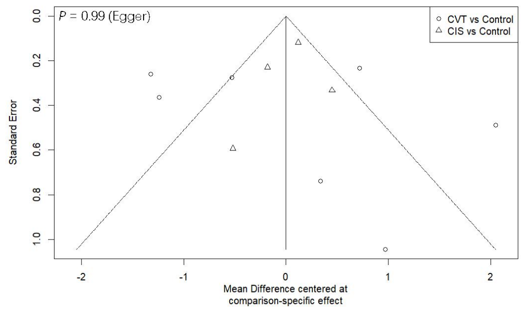

Neutrophil

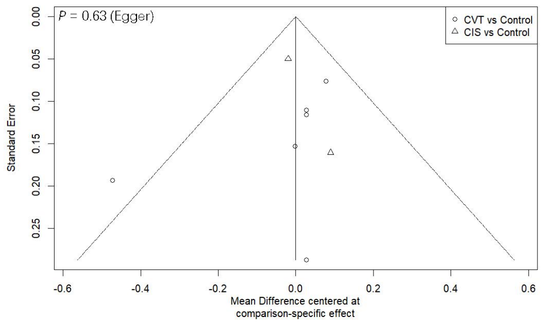

Lymphocyte

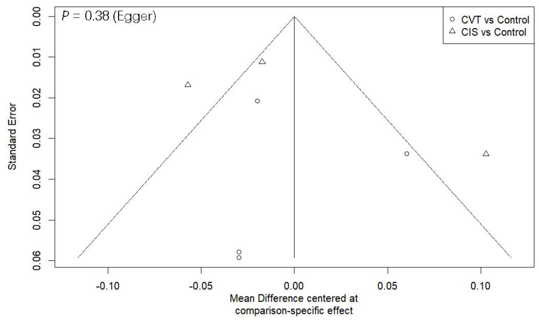

Monocyte

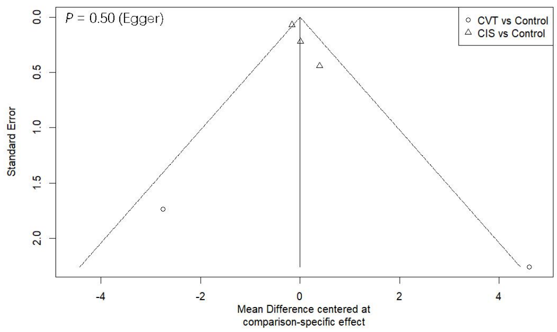

CRP

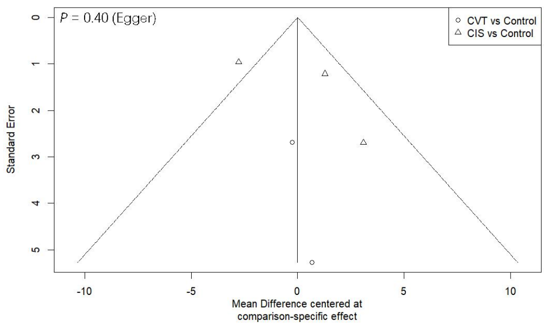

Hs-CRP

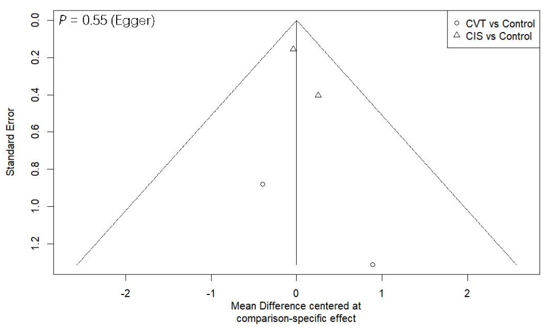

IL-6

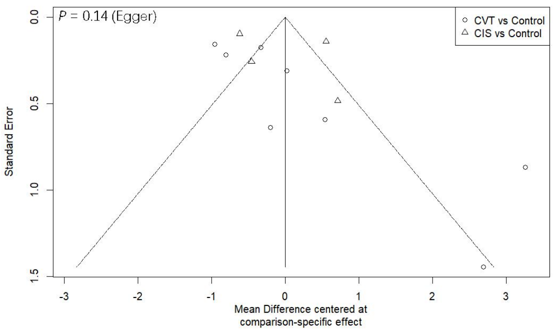

NLR

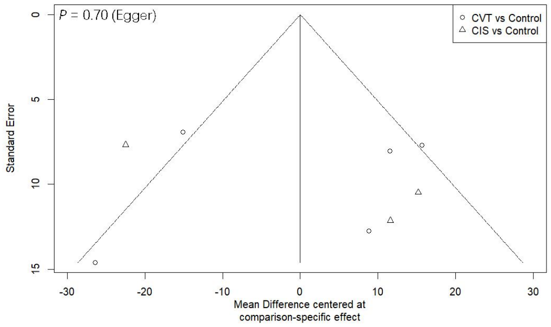

PLR

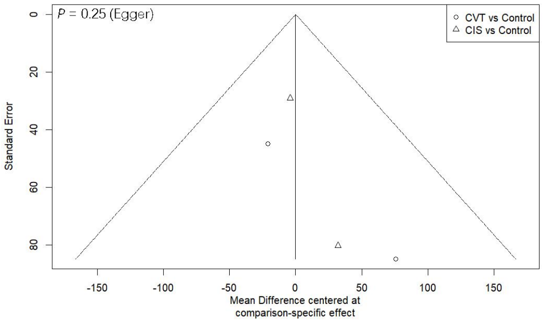

SII

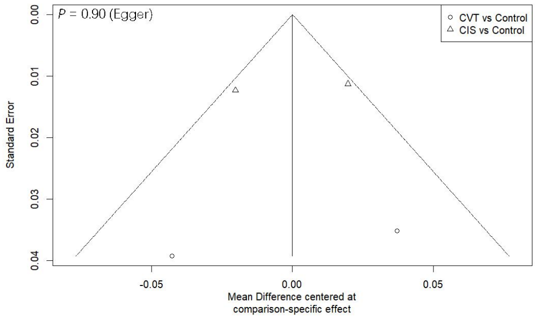

MHR

Figure S4. The adjusted funnel plot for the network meta-analysis comparing inflammatory markers between IS and CVT is symmetrical, and all Egger's tests exhibit P-values above the standard significance level (0.05), suggesting a low risk of publication bias in the included studies.

Figure S7: Convergence diagnostics of the network meta-analysis: comparison of

inflammatory markers between CVT and IS.

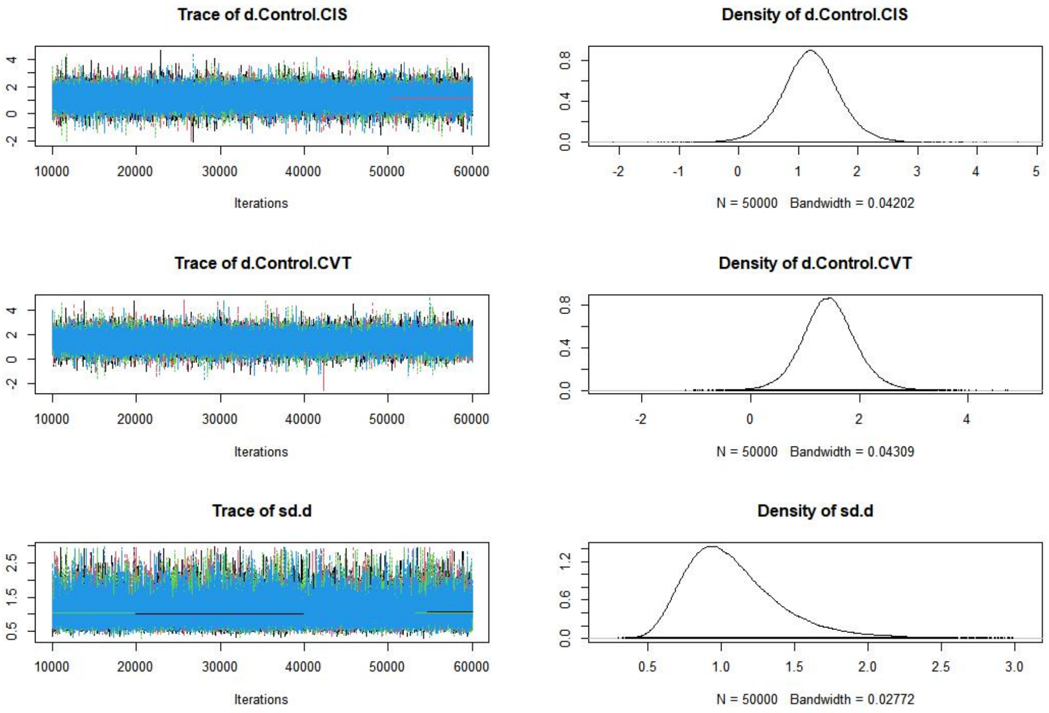

WBC

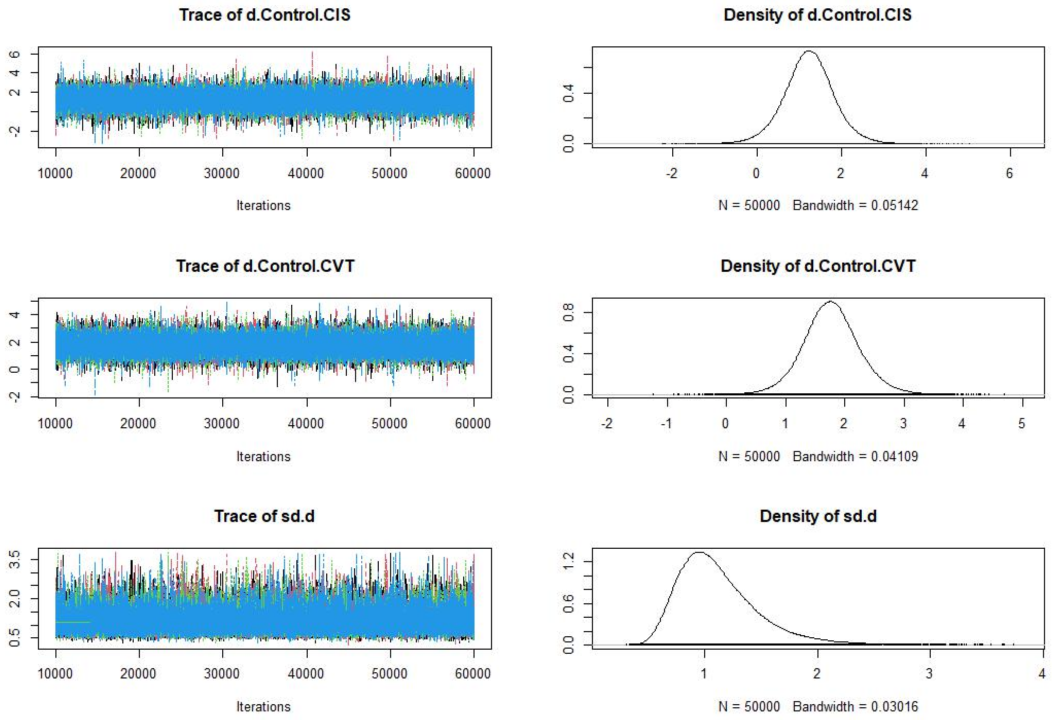

Neutrophil

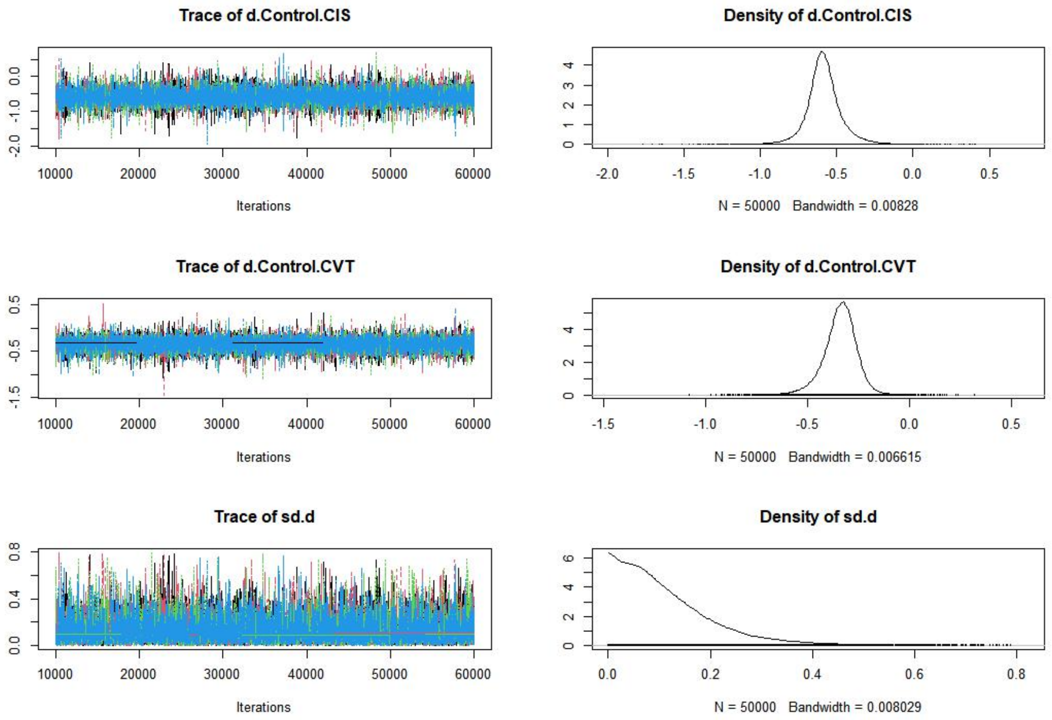

Lymphocyte

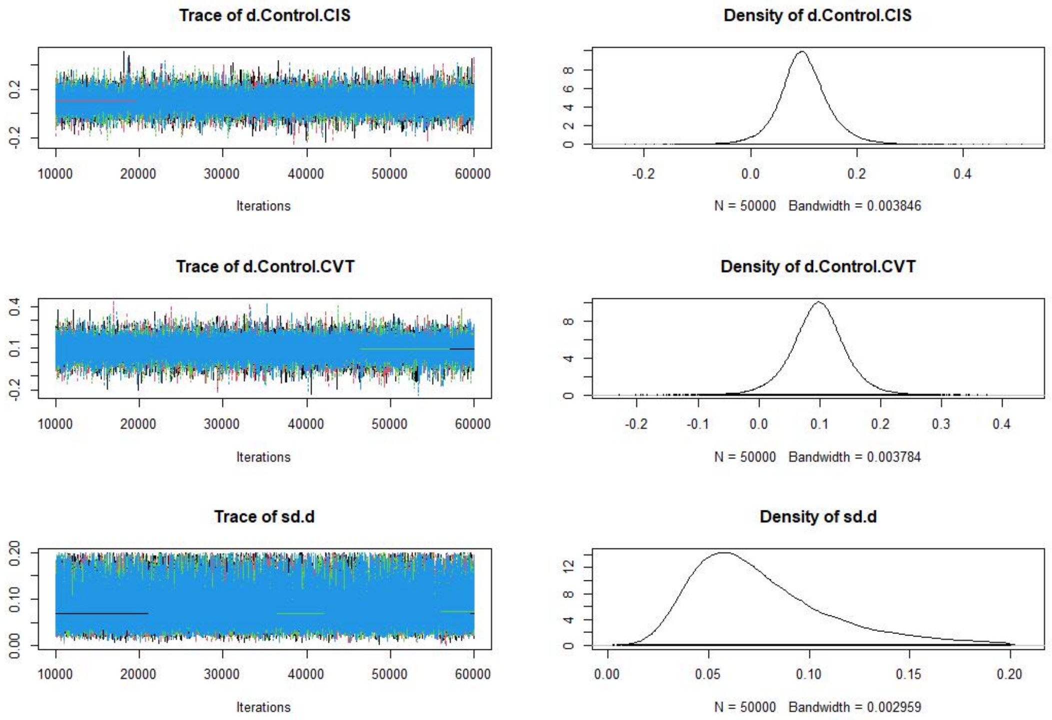

Monocyte

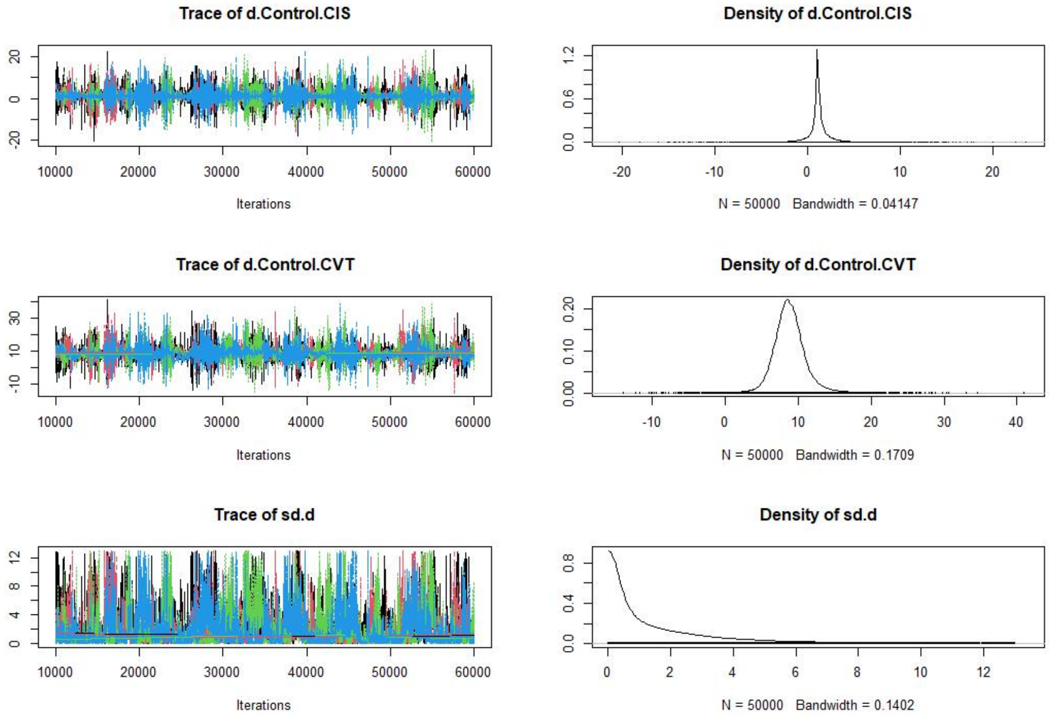
Trace of d.Control.CIS
Density of d.Control.CIS
Iterations
N = 50000 Bandwidth = 0.04147
Trace of d.Control.CVT
Density of d.Control.CVT
Iterations
N = 50000 Bandwidth = 0.1709
Trace of sd.d
Density of sd.d
Iterations
N = 50000 Bandwidth = 0.1402

CRP

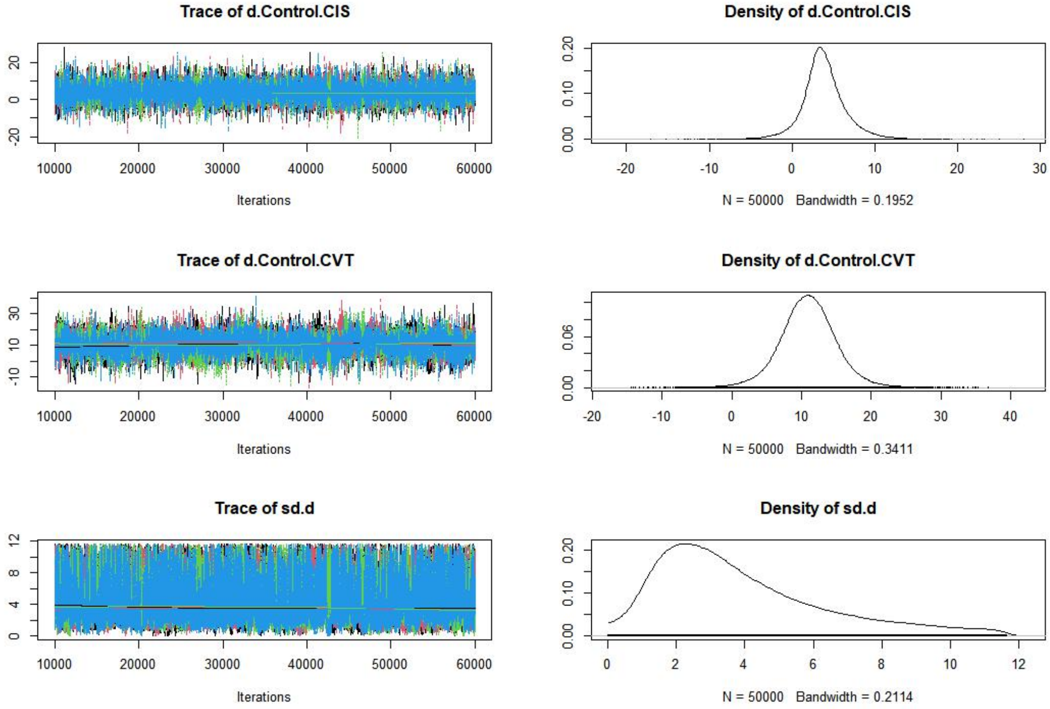
Trace of d.Control.CIS
Density of d.Control.CIS
Iterations
N = 50000 Bandwidth = 0.1952
Trace of d.Control.CVT
Density of d.Control.CVT
Iterations
N = 50000 Bandwidth = 0.3411
Trace of sd.d
Density of sd.d
Iterations
N = 50000 Bandwidth = 0.2114

Hs-CRP

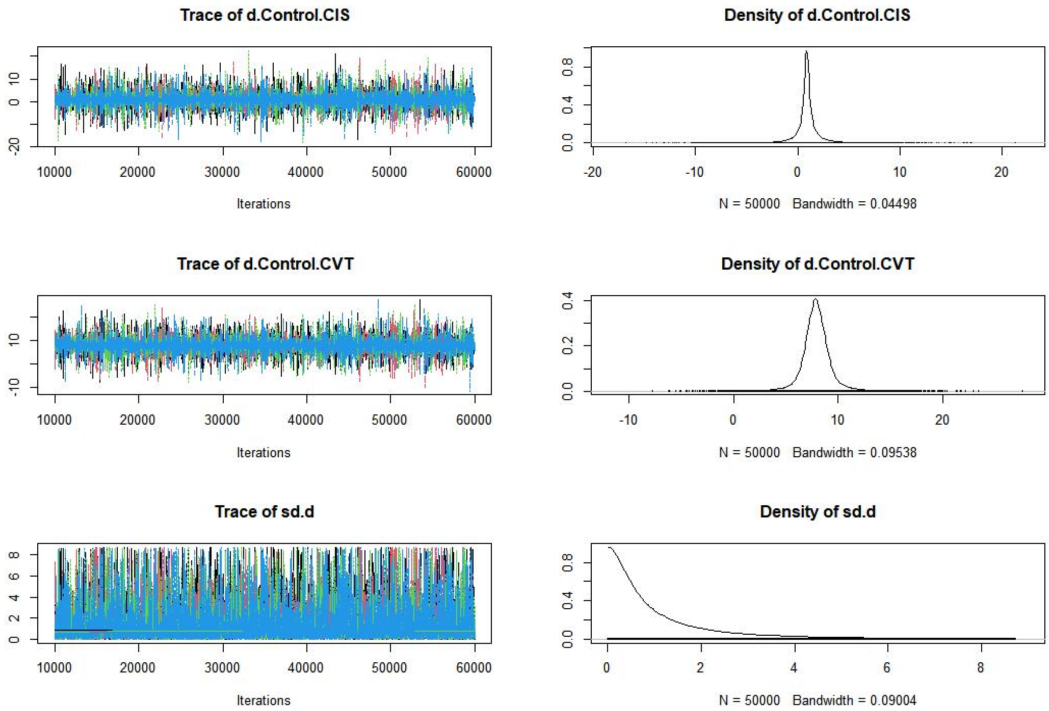
Trace of d.Control.CIS
Iterations
Density of d.Control.CIS
N = 50000 Bandwidth = 0.04498
Trace of d.Control.CVT
Iterations
Density of d.Control.CVT
N = 50000 Bandwidth = 0.09538
Trace of sd.d
Iterations
Density of sd.d
N = 50000 Bandwidth = 0.09004

IL-6

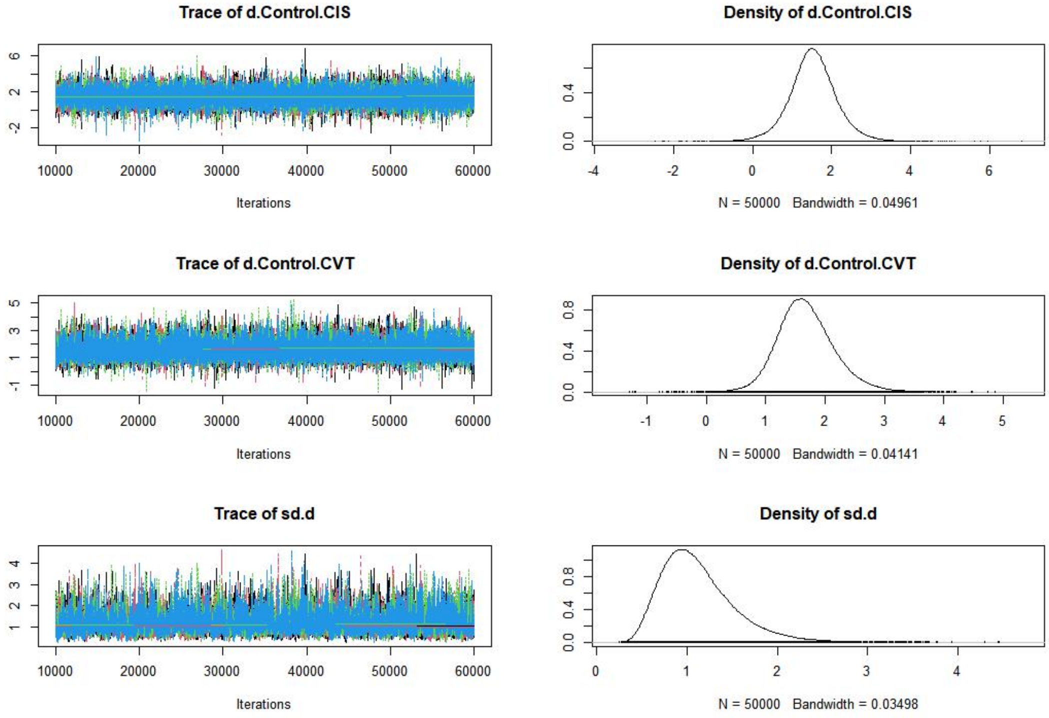
Trace of d.Control.CIS
Iterations
Density of d.Control.CIS
N = 50000 Bandwidth = 0.04961
Trace of d.Control.CVT
Iterations
Density of d.Control.CVT
N = 50000 Bandwidth = 0.04141
Trace of sd.d
Iterations
Density of sd.d
N = 50000 Bandwidth = 0.03498

NLR

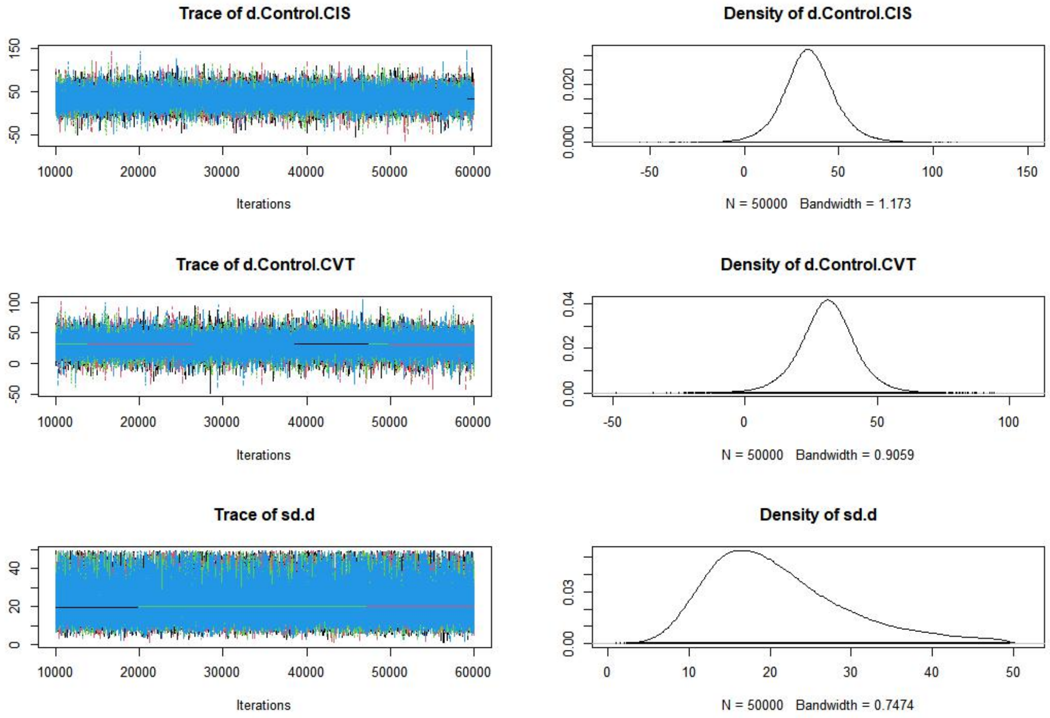
Trace of d.Control.CIS
Iterations
Density of d.Control.CIS
N = 50000 Bandwidth = 1.173
Trace of d.Control.CVT
Iterations
Density of d.Control.CVT
N = 50000 Bandwidth = 0.9059
Trace of sd.d
Iterations
Density of sd.d
N = 50000 Bandwidth = 0.7474

PLR

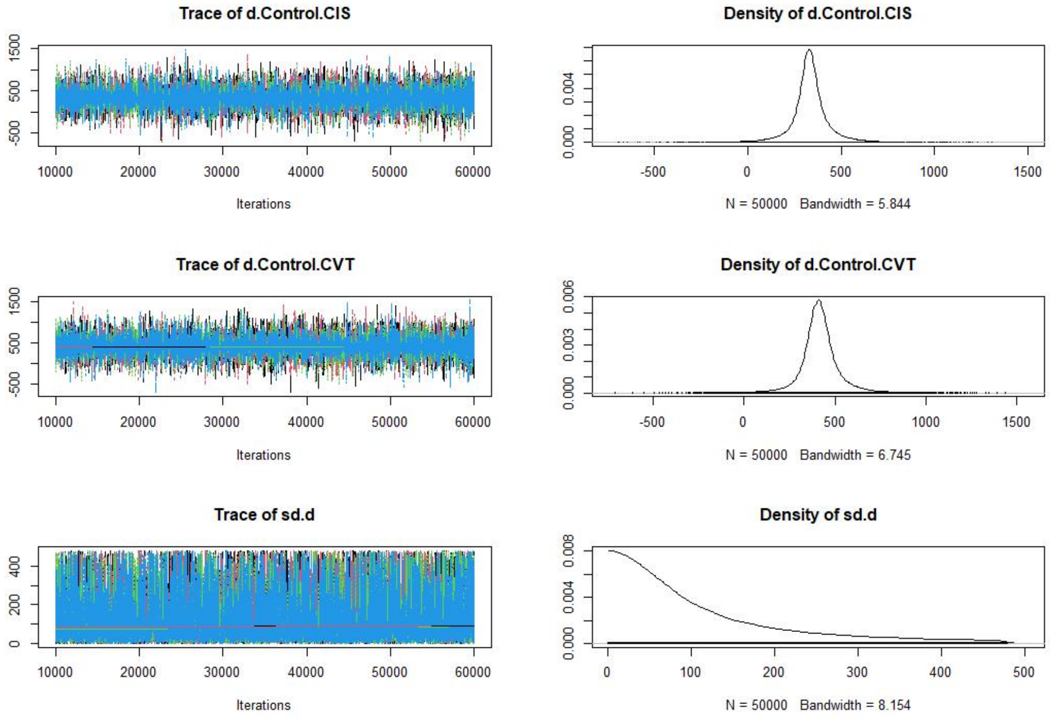
Trace of d.Control.CIS
Iterations
Density of d.Control.CIS
N = 50000 Bandwidth = 5.844
Trace of d.Control.CVT
Iterations
Density of d.Control.CVT
N = 50000 Bandwidth = 6.745
Trace of sd.d
Iterations
Density of sd.d
N = 50000 Bandwidth = 8.154

SII

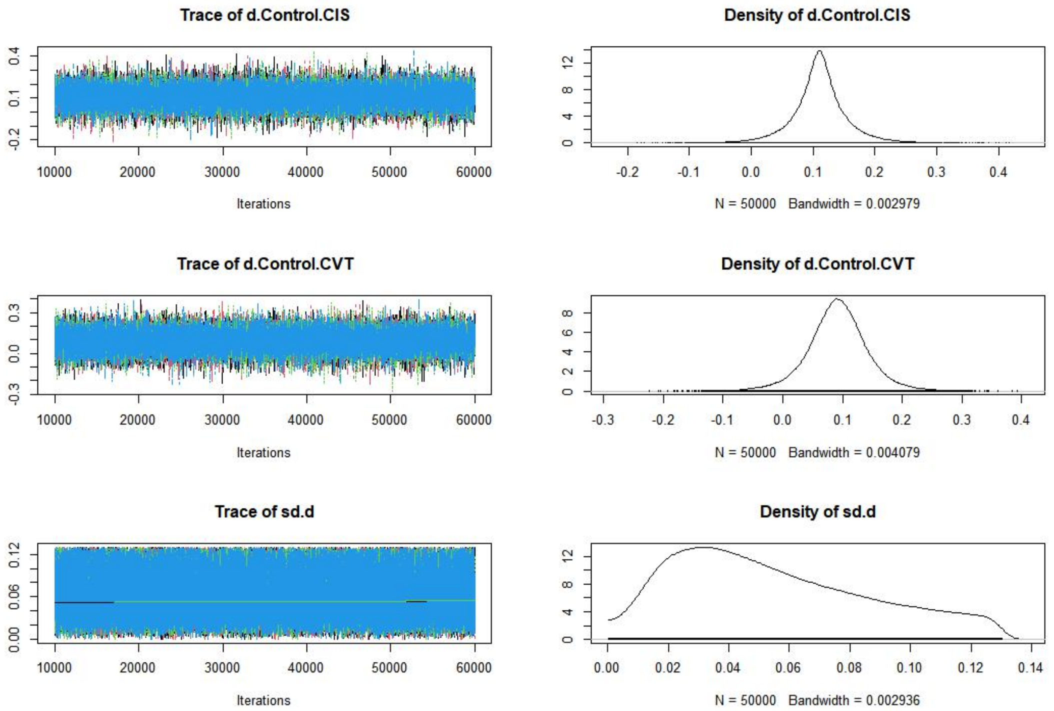

MHR

Abbreviations: CVT, Cerebral Venous Thrombosis; IS, Ischemic Stroke; WBC, White Blood Cell Count; Hs-CRP, High-sensitivity C-reactive Protein; CRP, C-reactive Protein; NLR, Neutrophil-to-Lymphocyte Ratio; SII, Systemic Immune-Inflammation Index; PLR, Platelet-to-Lymphocyte Ratio; MHR, Monocyte-to-High-Density Lipoprotein Cholesterol Ratio; IL-6, Interleukin-6.

Figure S8: Trace and density of the network meta-analysis: comparison of inflammatory markers between CVT and IS.

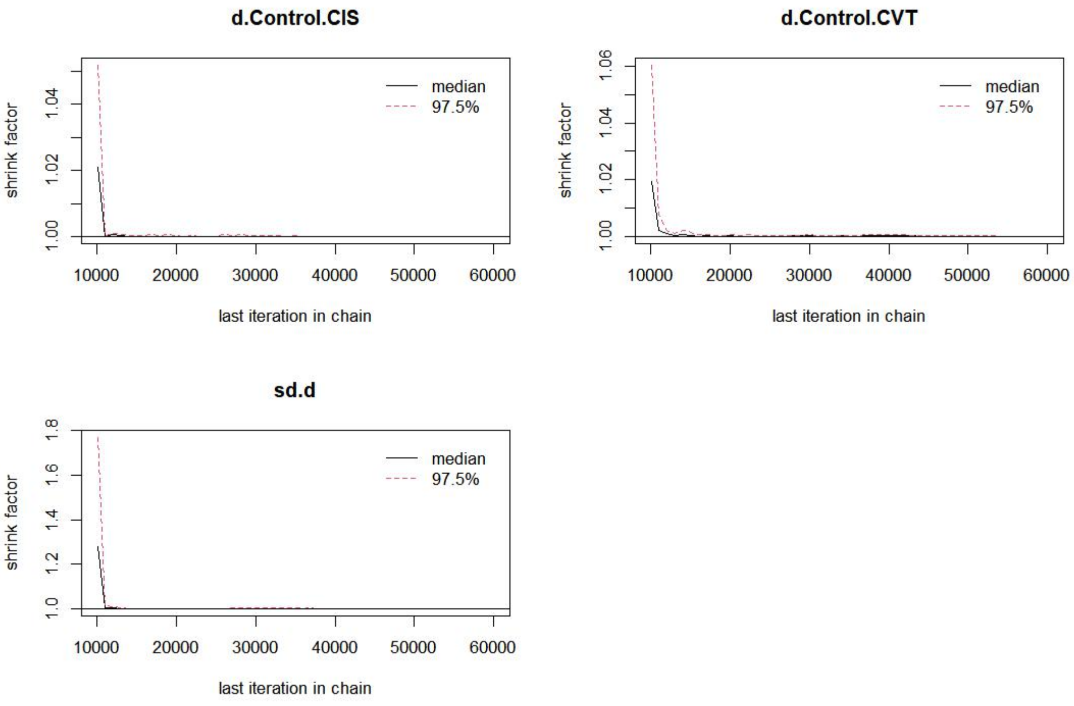

WBC

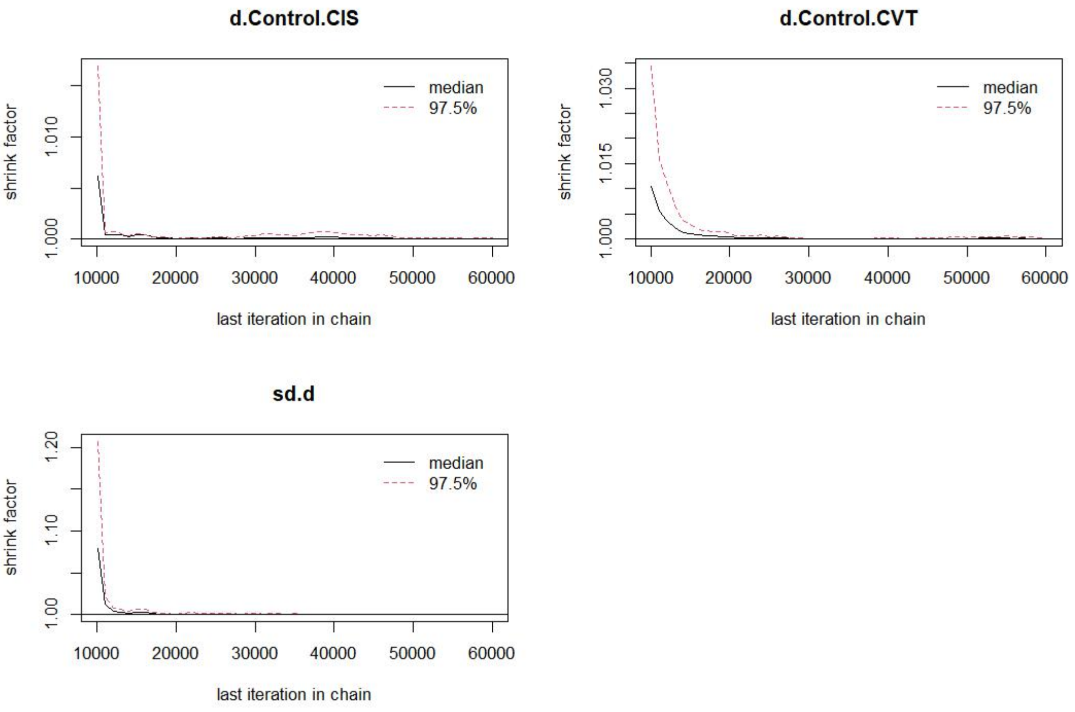

Neutrophil

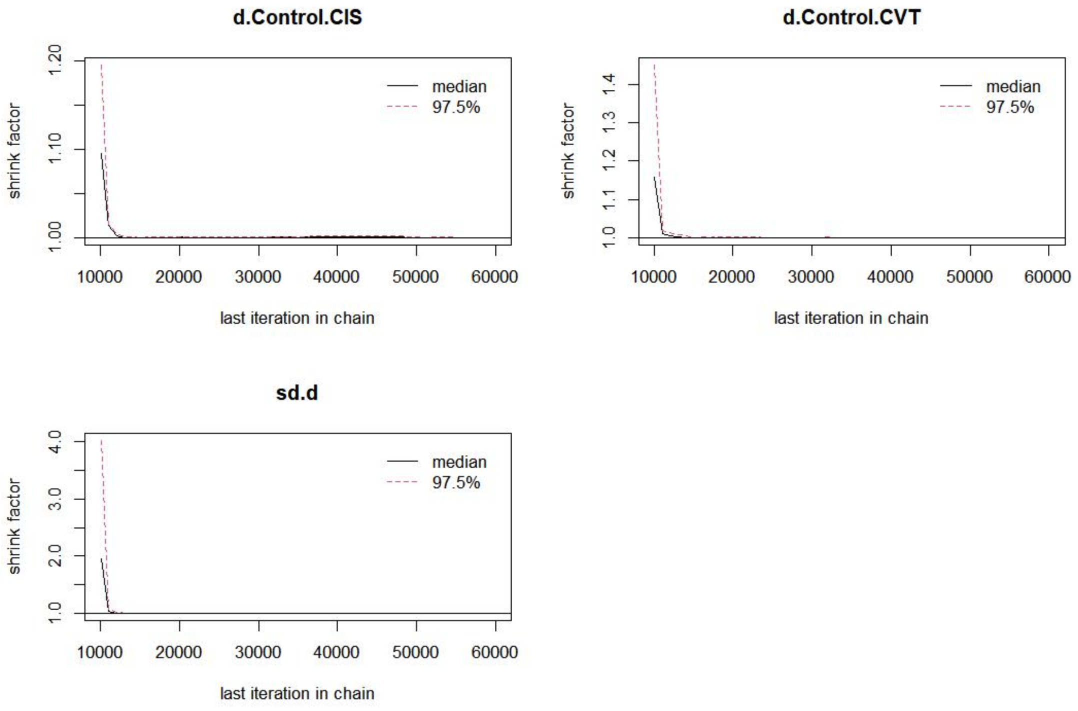

Lymphocyte

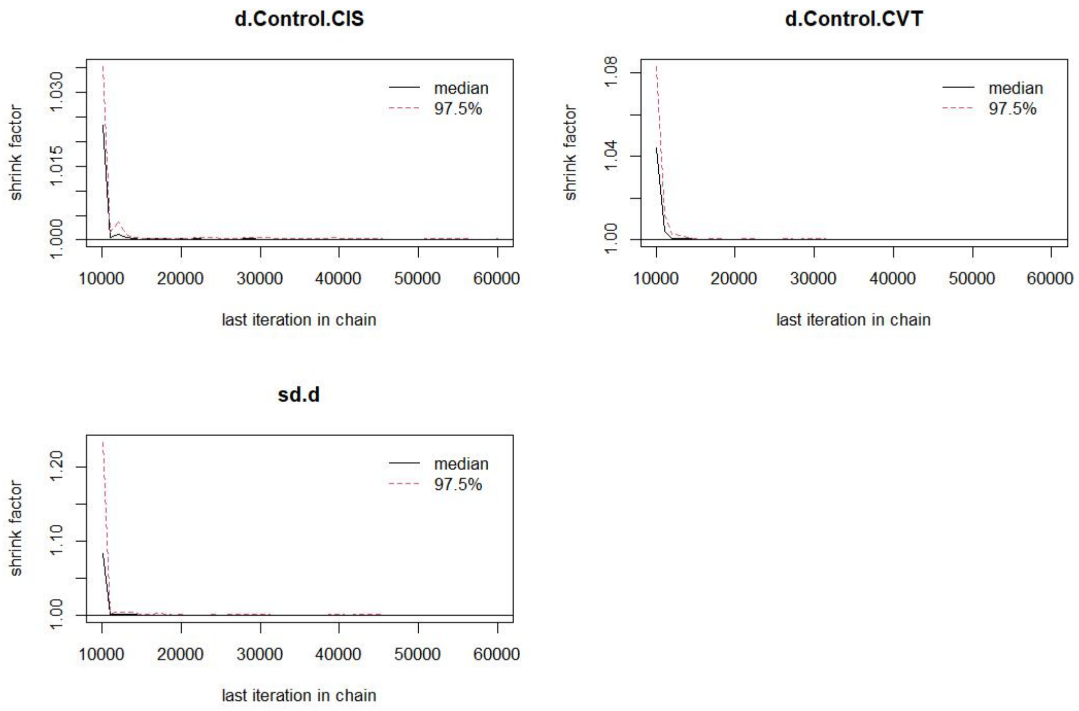

Monocyte

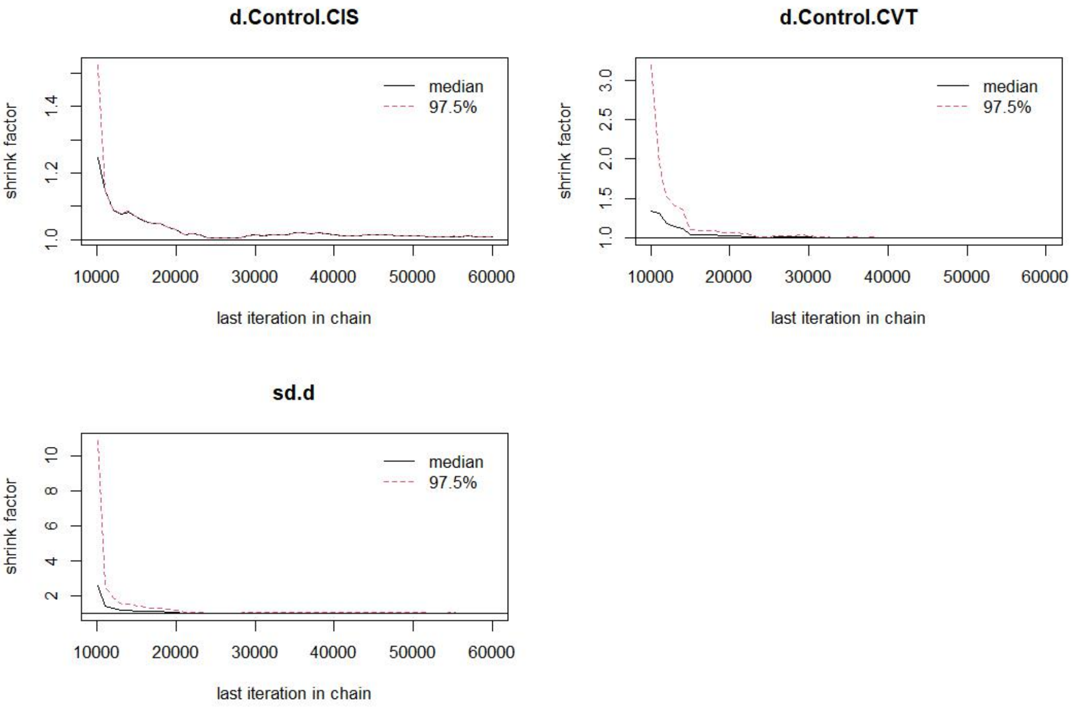

CRP

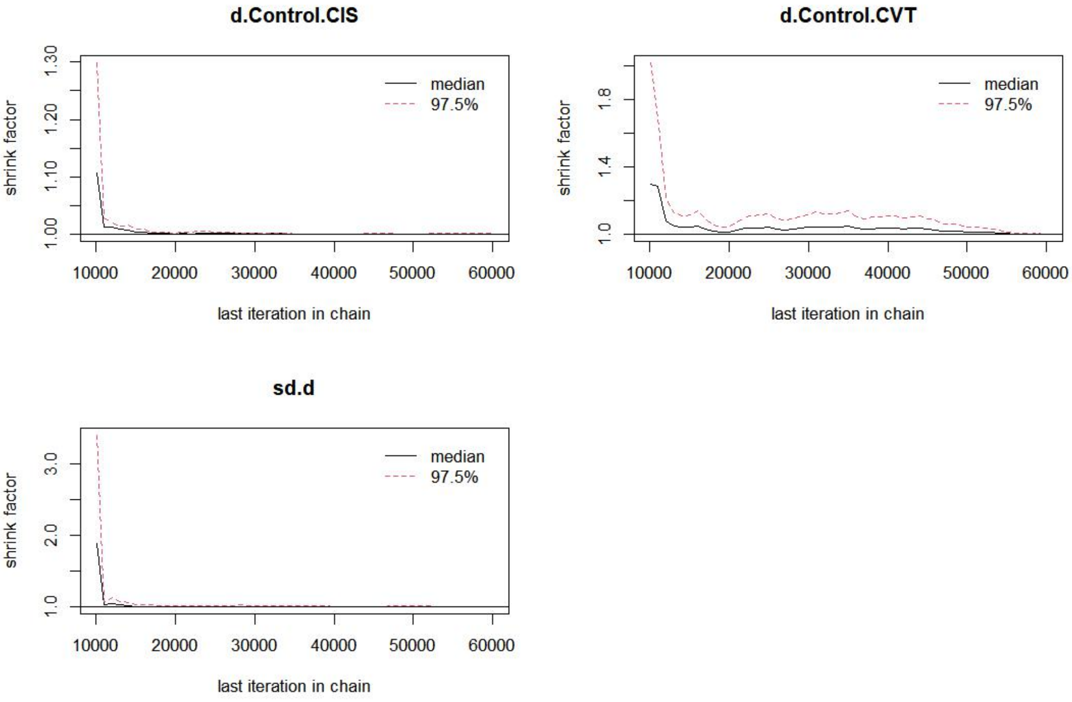

Hs-CRP

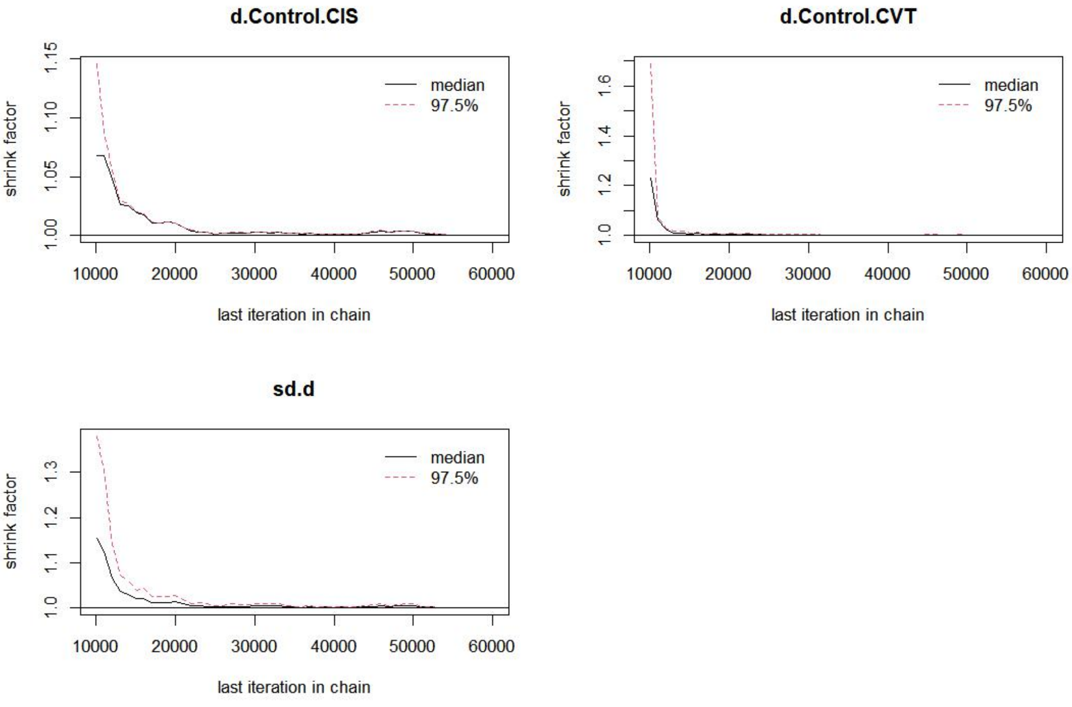
d.Control.CIS
d.Control.CVT
sd.d
median
97.5%
shrink factor
last iteration in chain
10000 20000 30000 40000 50000 60000

IL-6

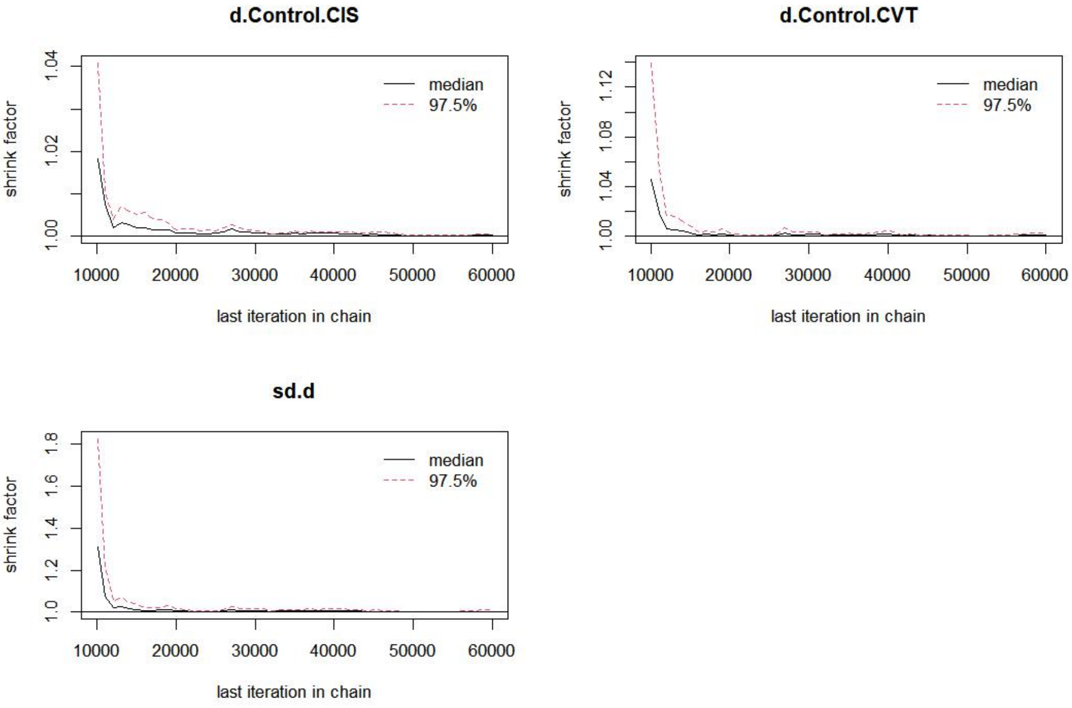
d.Control.CIS
d.Control.CVT
sd.d
median
97.5%
shrink factor
last iteration in chain
10000 20000 30000 40000 50000 60000

NLR

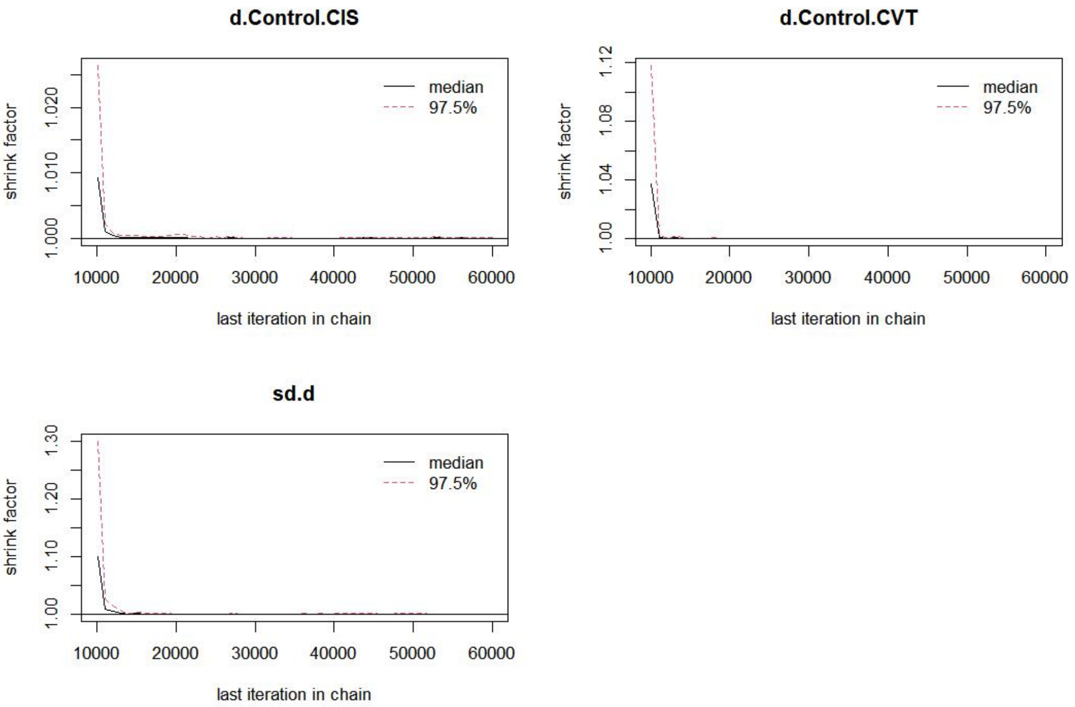
d.Control.CIS
d.Control.CVT
sd.d
shrink factor
last iteration in chain
median
97.5%
10000
20000
30000
40000
50000
60000

PLR

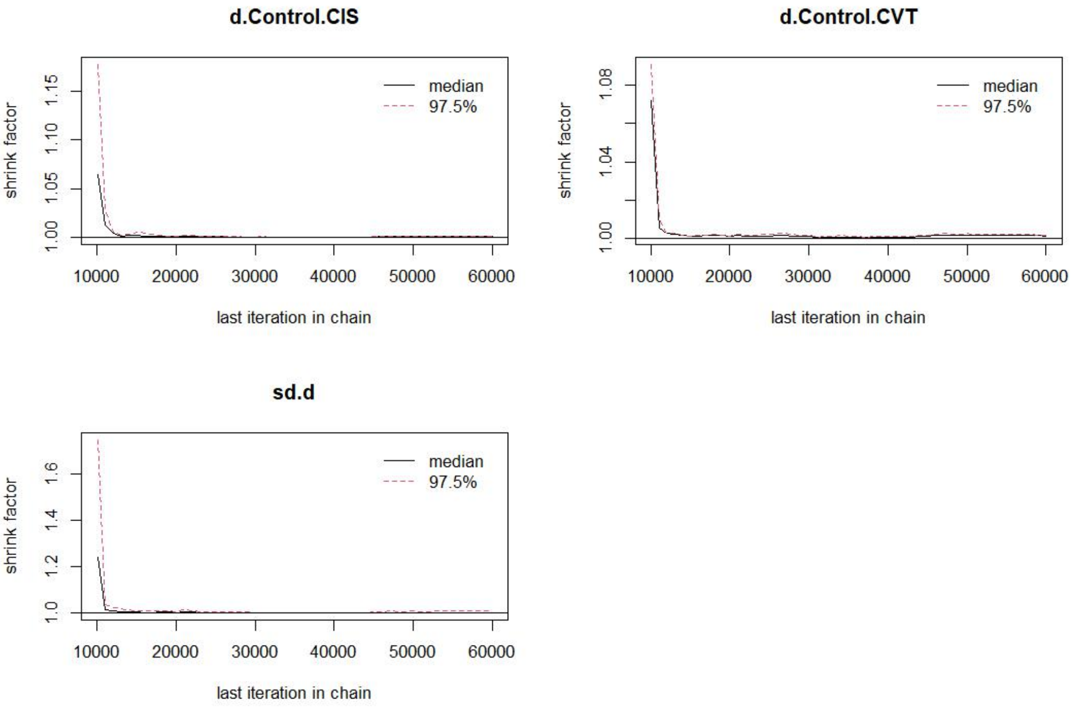
d.Control.CIS
d.Control.CVT
sd.d
shrink factor
last iteration in chain
median
97.5%
10000
20000
30000
40000
50000
60000

SII

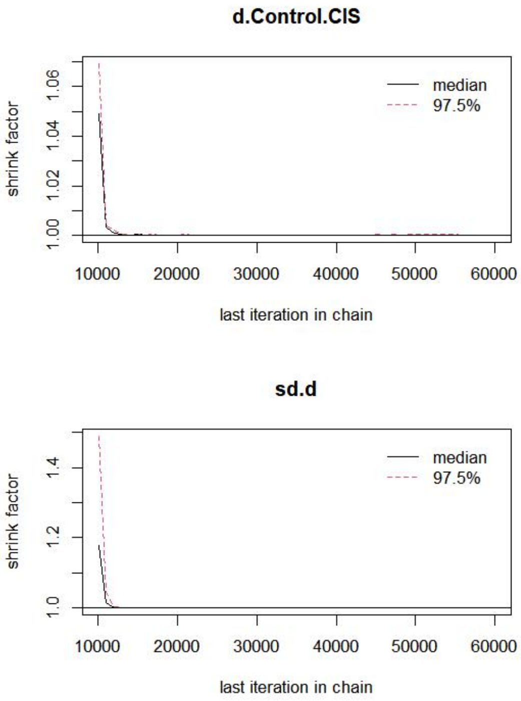

MHR

Abbreviations: CVT, Cerebral Venous Thrombosis; IS, Ischemic Stroke; WBC, White Blood Cell Count; Hs-CRP, High-sensitivity C-reactive Protein; CRP, C-reactive Protein; NLR, Neutrophil-to-Lymphocyte Ratio; SII, Systemic Immune-Inflammation Index; PLR, Platelet-to-Lymphocyte Ratio; MHR, Monocyte-to-High-Density Lipoprotein Cholesterol Ratio; IL-6, Interleukin-6.
